# Modulation of the gut microbiota by the mixture of fish oil and krill oil in high-fat diet-induced obesity mice

**DOI:** 10.1371/journal.pone.0186216

**Published:** 2017-10-09

**Authors:** Chenxi Cui, Yanyan Li, Hang Gao, Hongyan Zhang, Jiaojiao Han, Dijun Zhang, Ye Li, Jun Zhou, Chenyang Lu, Xiurong Su

**Affiliations:** 1 School of Marine Science, Ningbo University, Ningbo, Zhejiang, China; 2 Department of Food Science, Cornell University, New York, NY, United States of America; 3 The First Bethune Hospital of Jilin University, Changchun, Jilin, China; University of Missouri Columbia, UNITED STATES

## Abstract

Previous studies confirmed that dietary supplements of fish oil and krill oil can alleviate obesity in mice, but the underlying mechanism remains unclear. This study aims to discern whether oil treatment change the structure of the gut microbiota during the obesity alleviation. The ICR mice received high-fat diet (HFD) continuously for 12 weeks after two weeks of acclimatization with a standard chow diet, and the mice fed with a standard chow diet were used as the control. In the groups that received HFD with oil supplementation, the weight gains were attenuated and the liver index, total cholesterol, triglyceride and low-density lipoprotein cholesterol were reduced stepwise compared with the HFD group, and the overall structure of the gut microbiota, which was modulated in the HFD group, was shifted toward the structure found in the control group. Moreover, eighty-two altered operational taxonomic units responsive to oil treatment were identified and nineteen of them differing in one or more parameters associated with obesity. In conclusion, this study confirmed the effect of oil treatment on obesity alleviation, as well as on the microbiota structure alterations. We proposed that further researches are needed to elucidate the causal relationship between obesity alleviation and gut microbiota modulation.

## Introduction

In the last three decades, overweight and obesity levels have more than doubled worldwide. In 2014, 39% of 18-year-olds in the world were overweight, and 13% were obese [1]. Accumulating evidence suggests that lipocytes not only provide energy storage but also act as an integral part of the endocrine function of superfluous fat tissue, with diverse health consequences [2]. Obesity often contributes to the pathogenesis, complicates the course, and increases the risk of several other life-threatening diseases [3]. Obesity-related metabolic diseases have attracted great attention all over the world. The etiology, prevention and treatment of such problems has become a global research interest in the past few decades [4].

The development of obesity is a complicated process involving genetic and environmental factors. High energy intake and low physical activity are the typical environmental factors associated with obesity. Obesity usually accompanied with increase of total cholesterol (TC), triglyceride (TG) and low-density lipoprotein cholesterol (LDL-C) and a decrease of high-density lipoprotein cholesterol (HDL-C) in serum [5]. These four indicators had been widely used to validate obesity symptoms in various studies related to obesity [6–9]. Several pharmacotherapeutic agents, such as phentermine, have been employed to alleviate this complex disorder in recent decades. They mainly exert their effects by restraining appetite, controlling lipometabolism, and restricting energy intake [10–12]. However, the development of pharmacological drugs has been seriously challenged because candidate drugs tended to lack efficacy and have various side effects, such as depression, suicide and apoplexy [13]. The regulatory agencies has limited the use of pharmacological agents and removed some drugs from the market [14]. Therefore, intervention strategies with new targets are needed to advance the treatment of obesity.

Accumulating evidence suggests that the obesity and its complications such as insulin resistance, hyperlipidemia and atherosclerosis caused by high-fat, high calorie diet are often accompanied with the alteration in the gut microbiota [15, 16]. The ratio of *Firmicutes* to *Bacteroidetes* is the best known example at the phylum level, which increased in obese mice when compared with lean ones [17]. Furthermore, the development of obesity, adipose tissue and systemic inflammation and metabolic comorbidities in humans are also associated with the decreased in probiotics (such as *Bifidobacterium* spp. and *Pediococcus pentosaceus*) and increased in pro-inflammatory/pathogenic bacteria (such as *Desulfovibrionaceae*) [18, 19]. As special species in the gut microbiota, such as *Enterobacter cloacae*, *Akkermansia muciniphila*, *Clostridium bolteae* and *Desulfovibrio* sp, were proved to be involved in the development of obesity and its-related metabolic diseases, such as type 2 diabetes [18, 20]. Up to now, there is still lack of studies showed that the gut microbiota acted as a pivotal contributing factor in the development of obesity, whereas some studies found that fat deposits was increased in germ-free mouse recipients after transplantation of the gut microbiota from obese humans or mice [21, 22]. These studies suggest that intestinal microbiota might make contribution to metabolic syndrome therapy, and it may be a potential target for disease controlling.

Omega-3 (Ω-3) fatty acids, which are abundant in fatty fish, marine plankton, walnut oil and linseed (flaxseed) oil, rank among the most important essential nutrients [23]. The consumption of Ω-3 fatty acids, especially docosahexaenoic acid (DHA) and eicosapentaenoic acid (EPA), is strongly correlated with health benefits. A previous study showed that fish oil supplementation (1 g/kg/day) was able to reduce TC and TG levels, in addition to improved systemic and muscle insulin sensitivity in rat [24]. Furthermore, fish oil supplementation ameliorated some inflammatory diseases such as asthma and Crohn’s disease [25, 26]. Krill oil is extracted from Antarctic krill. It contains long-chain Ω-3 polyunsaturated fatty acids [27]. The fatty acids in fish oil are stored as triglyceride, whereas in krill oil approximately 30–65% of the fatty acids are incorporated into phospholipids [28]. In addition, krill oil contains astaxanthin, and it may maintain the stability of EPA and DHA against oxidative damage [29]. Various studies have confirmed that supplementation with krill oil alleviated chronic disorders, such as cardiovascular diseases, endocannabinoid dysregulation, poor infant development, non-alcoholic fatty liver disease, premenstrual syndrome, inflammation and certain cancers [28, 30]. The beneficial effects of krill oil and fish oil on human health have been confirmed. However, there is no direct evidence that krill oil and fish oil alleviate obesity or other metabolic syndromes by modulating the gut microbiota, and the efficacy of mixtures of the two oils against obesity remains unclear.

For the study, we used a randomized approach to assess the efficacy of pure oils or various mixtures thereof against obesity in mice. The blood biochemical indices, the liver index, and the shift in the structure of the gut microbiota in response to different ratios of oil supplementation in obese mice were examined. Our results provide new insight into the alteration of gut microbiota after the oil treatment in the high fat diet induced obesity mice, and this study may help to identify alternative strategies to ameliorate obesity.

## Materials and methods

### Ethics statement

All experimental and animal care procedures were performed in accordance with the guideline prepared by the Ningbo University Laboratory Animal Center (affiliated with the Zhejiang Laboratory Animal Common Service Platform), and all of the protocols were approved by the Ningbo University Laboratory Animal Center under permit number SYXK (ZHE 2008–0110).

### Animal trial

All experimental procedures and animal care were in accordance with the experimental animal care and use guidelines prepared by the Ningbo University Experimental Animal Center (affiliated with the Zhejiang Laboratory Animal Common Service Platform), and all animal programs have received the approval of the Ningbo University Laboratory Animal Center under permit number No. SCXK (ZHE 2014–0001).

After 2 weeks of acclimatization with a standard chow diet, we randomly divided 96 10-week-old male ICR mice (23.33±2.17 g, purchased from Laboratory Animal Center of Zhejiang province (Hangzhou, China), SCXK (Zhejiang) 2014–0001) into 8 groups (12 mice per group): (1) control group, the mice in control group was fed normal chow (protein with 20% Kcal, carbohydrate with 70% kcal, fat with 10% kcal, purchased from Laboratory Animal Center of Ningbo University, Ningbo, China); (2) HFD group, The mice in high-fat diet (HFD) group was fed with high-fat diet (protein with 20% Kcal, carbohydrate with 35% kcal, fat with 45% kcal purchased from Laboratory Animal Center of Ningbo University, Ningbo, China); (3) HFD+M group, fed with HFD and received 1 μg/g metformin by gavage once daily; (4) HFD+FO600 group, fed with HFD and received 600 μg/g fish oil by gavage once daily; (5) HFD+KO600 group, fed with HFD and received 600 μg/g krill oil by gavage once daily; (6) HFD+FO300KO300 group, fed with HFD and received 600 μg/g mixture of fish oil and krill oil in a ratio of 1:1 by gavage once daily; (7) HFD+FO400KO200 group, fed with HFD and received 600 μg/g mixture of fish oil and krill oil in a ratio of 2:1 by gavage once daily; (8) HFD+FO450KO150 group, fed with HFD and received 600 μg/g mixture of fish oil and krill oil in a ratio of 3:1 by gavage once daily. All oil and drug were given to mice by gavage in 0.9% stroke-physiological saline solution. Non-treatment groups were given the same volume of 0.9% stroke-physiological saline solution to minimize the effects of gavage procedure. Within each group, the twelve mice were divided into three cages (4 mice in a cage). The mice were kept in the same house during the experiments simultaneously for 12 weeks, and none of the mice died.

Throughout the duration of the trial, the body weight of each mouse was monitored weekly. At the end of the trial, fecal samples were collected and placed in liquid nitrogen immediately after sampling and stored at -80°C. After 12 h of food deprivation, fasting body weight was precisely examined and then all the animals were anaesthetized with ether. Ether was used as the anesthetic due to its high efficiency, low cost and easy manipulation, thus it was still widely used in the animal experiments [7, 31, 32]. In consideration that the use of ether as an anesthetic might pose a safety hazard to researchers, a similar small equipment as previously described had been made and used to reduce the safety hazard [33]. Blood was collected from the orbital plexus, and serum was isolated by centrifugation at 3000 rpm at 4°C for 15 min and stored at -80°C for subsequent biochemical testing. Then, mice were sacrificed by cervical dislocation, and the tissues, including adipose tissues (epididymal, subcutaneous, visceral, interscapular) and liver were excised, weighed, and frozen in liquid nitrogen immediately for further analysis. Adiposity index was then calculated according to the following formula: adiposity weight/body weight (mg/g). The liver index was calculated using this formula: liver weight/body weight (mg/g).

### Analysis of physiological and biochemical indices in plasma

TC, TG, LDL-C and HDL-C levels in the plasma were measured using commercial enzymatic kits purchased from Nanjing Jiancheng Bioengineering Institute (Nanjing, China) according to the manufacturer's instructions.

### Measurement of the composition of fish oil and krill oil

The fatty acid composition of krill oil and fish oil was measured via Gas Chromatography-Mass Spectrometer (GC-MS) as previously described [34]. The concentration of astaxanthin in krill oil was measured via High Performance Liquid Chromatography (HPLC) as previously described [35] and the astaxanthin standard was purchased from Sigma-Aldrich Co., LLC(St. Louis, MO, USA).

### Total DNA extraction, PCR and sequencing

The total DNA was isolated from each sample (feces from a shared cage) using a previously described method [36]. The quantity of extracted DNA was measured using a Thermo NanoDrop 2000C (Thermo Fisher Scientific, USA).

PCR primers were designed in the V3 and V4 hypervariable regions of the bacterial 16S rRNA gene. The primers 319F 5′-ACTCCTACGGGAGGCAGCAG-3′ and 806R 5′-GGACTACHVGGGTWTCTAAT-3′ were designed with a barcode sequence that was unique to each sample. PCR products which satisfied the quality demands were used for further sequencing. Amplification reactions were performed in 25 μL volume containing 20 ng of template, 0.1 μM of each primer and 12.5 μL Premix Ex TaqTM Hot Start Version (Takara Biotechnology Co. Ltd, Dalian, China). Amplification was initiated at 98°C for 30 s, followed by 35 cycles of denaturation at 98°C for 10 s, primer annealing at 54°C for 30 s, extension at 72°C for 45 s, and final extension for 10 min. PCR reactions for each sample were performed with a negative control in each run. The presence of amplicons was confirmed using gel electrophoresis, and the PCR products were normalized using AxyPrepTM Mag PCR Normalizer and then sequenced using the MiSeq system constructed with the Illumina Nextera XT Index kit in Sangon Biotech Co., Ltd. (Shanghai, China). Sequencing was performed on an Illumina MiSeq (Illumina, San Diego, CA, USA) using 2×300 bp paired-end sequencing and multiple sequencing runs in accordance with the manufacturer’s instructions.

### Data and statistical analysis

Raw FASTQ files were multiplexed and filtered using QIIME[37] (version 1.8.0) according to the following steps: (1) the reads that overlapped more than 10 bp were merged via software FLASH [38], and the maximum of overlap length was 70 bp and the ratio of sequence mismatches was 0.25. Reads that could not be merged were removed. (2) The data belonging to each sample were identified using the barcode sequence. (3) The reads were truncated at any position with an average quality score below 20 in a 10 bp sliding window. (4) Reads that contained undetected nucleotides (N) or were shorter than 200 bp were removed. UCHIME (version 4.2.40) [39] was used to identify and remove the chimeric sequences. Operational taxonomic units (OTUs) were clustered using USEARCH (version 7.1) [40], and 97% was chosen as the similarity level in OTU analysis. The community richness and community diversity indices were calculated using QIIME (version 1.8.0) and sequence normalized to the smallest number to reduce biases in sequencing depth for alpha and beta diversity analysis [41]. Taxonomic classifications were identified via the Ribosomal Database Project Classifier [42].

All data are represented as the means ± S.D. ANOVA and Tukey's post hoc test (SPSS, veision 19.0, Chicago, IL, USA) were used to analyze the normally distributed data, and the Mann-Whitney test (MATLAB R2012a, Natick, MA, USA) was used to analyze the data that did not meet the assumptions of ANOVA. *P*<0.05 was defined as a standard criterion of statistical significance. Correlations between the intestinal microbial composition and the phenotype of hyperlipidemia were calculated via Spearman’s correlation (SPSS) with Spearman’s correlation coefficient (R) and P-value. Correlations were considered as significant when *P*<0.05, and correlations were significant at false discovery rate<0.25.

### Data availability statement

The sequences have been deposited in the NCBI Sequence Read Archive database under the accession number SRP101547.

## Results

### Measurement of the composition of krill oil and fish oil

The fatty acid compositions of the krill oil and fish oil are presented in S1 Table. The concentrations of unsaturated fatty acids (FA) in krill oil and fish oil are 69.46% and 64.98%, respectively. The fish oil contained 25.76% DHA and 5.67% EPA, while the krill oil contained 3.90% DHA and 17.82% EPA. The astaxanthin concentration was 44.98 mg/100 g in the krill oil.

### Effect of oil supplementation on body weight gain, liver index and fat pad weights

**Fig 1:**
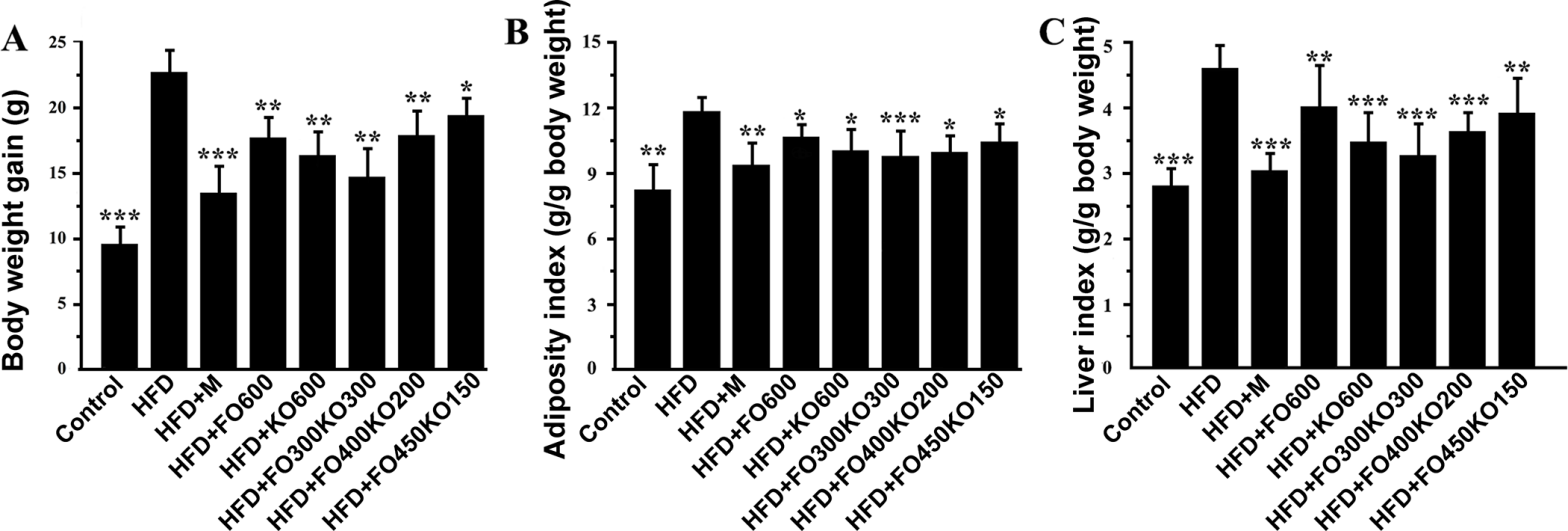
Effect of oil supplementation on physical features in HFD-induced mice. (A) Relative body weight gain. (B) Adiposity index. (C) Liver index. Data are represented as the means ± S.D. ***P<0.001, **P<0.01 and *P<0.05 vs the HFD group by ANOVA followed by Tukey post hoc test.

A high-fat (HFD) diet induced obesity in mice after 12 weeks of feeding (S2 Table). Fish oil, krill oil or their mixture was gavaged for 12 weeks while the animals received HFD feed. All of these oil treatments attenuated the increase of body weight. Likewise, adiposity index and liver index were significantly enhanced in the HFD group compared with the control (*P*<0.01). When compared to the HFD group, oil supplementation reduced the adiposity index and liver index of all the groups (Fig 1). The HFD+M group showed the same pattern as the oil groups in terms of the body weight gain, adiposity index and liver index. In addition, HFD+FO300KO300 showed significant efficacy in alleviating body weight gain and decreasing the adiposity index and liver index (*P*<0.01).

### Effect of oil supplementation on plasma biochemical indices

**Fig 2:**
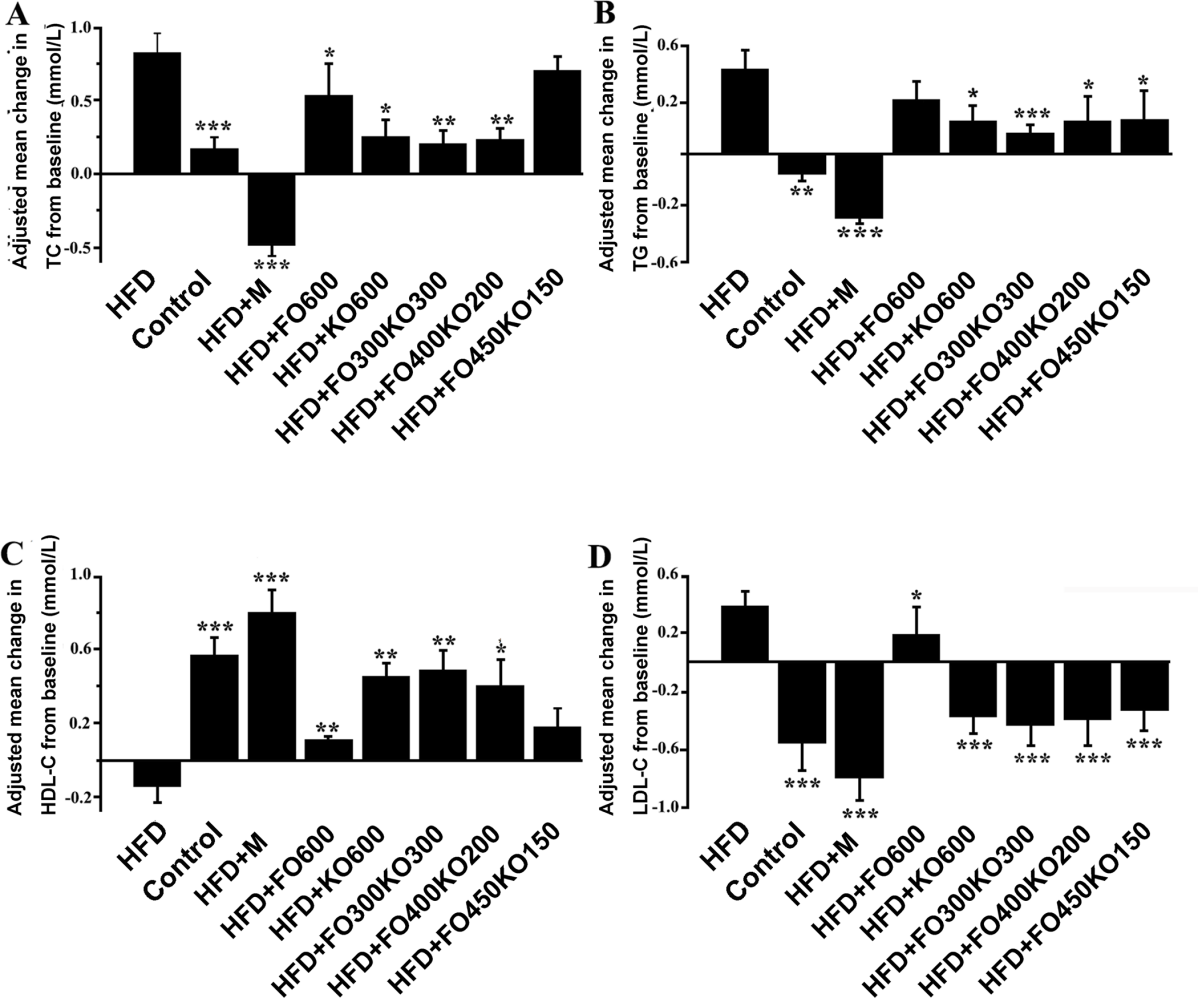
Effect of supplementation with oil on plasma biochemical indicators of mice fed an HFD. (A) The level of TC. (B) The level of TG. (C) The level of HDL-C. (D) The level of LDL-C. TC: total cholesterol, TG: triglyceride, HDL-C: high-density lipoprotein cholesterol, LDL-C: low-density lipoprotein cholesterol. Data are represented as the means ± S.D. ***P<0.001, **P<0.01 and *P<0.05 vs the HFD group by ANOVA followed by Tukey post hoc test.

HFD-fed mice developed the hallmark features of obesity and revealed significant increases from baseline in the adjusted means of LDL-C (*P*<0.001), TC (*P*<0.001) and TG (*P*<0.001) compared with the control group. Compared with the control group, a decrease in HDL-C (*P*<0.001) was also observed in HFD-fed mice. Oil treatment significantly attenuated obesity in HFD-fed mice. The HFD+FO300KO300 group showed significant reductions in LDL-C (*P*<0.001), TC (*P*<0.01) and TG (*P*<0.001) and a significant enhancement in HDL-C (*P*<0.01) compared with the HFD group (Fig 2 and S3 Table).

### Quality control and microbial diversity

To elucidate the effects of the oil treatment on the gut microbiota, we collected fecal samples from 96 mice after 12 weeks and performed 16S amplicon sequencing analyses of the V3 and V4 regions (S4 Table). The Simpson and Shannon diversity indices were used to measure the diversity of the microbial communities in each group. A diversity index is used to represent the number of different taxa in the sample, a higher Shannon index indicates greater diversity. According to these metrics, an HFD significantly reduced the diversity of the gut microbiota in mice, while oil treatment restored the index (S5 Table).

**Fig 3:**
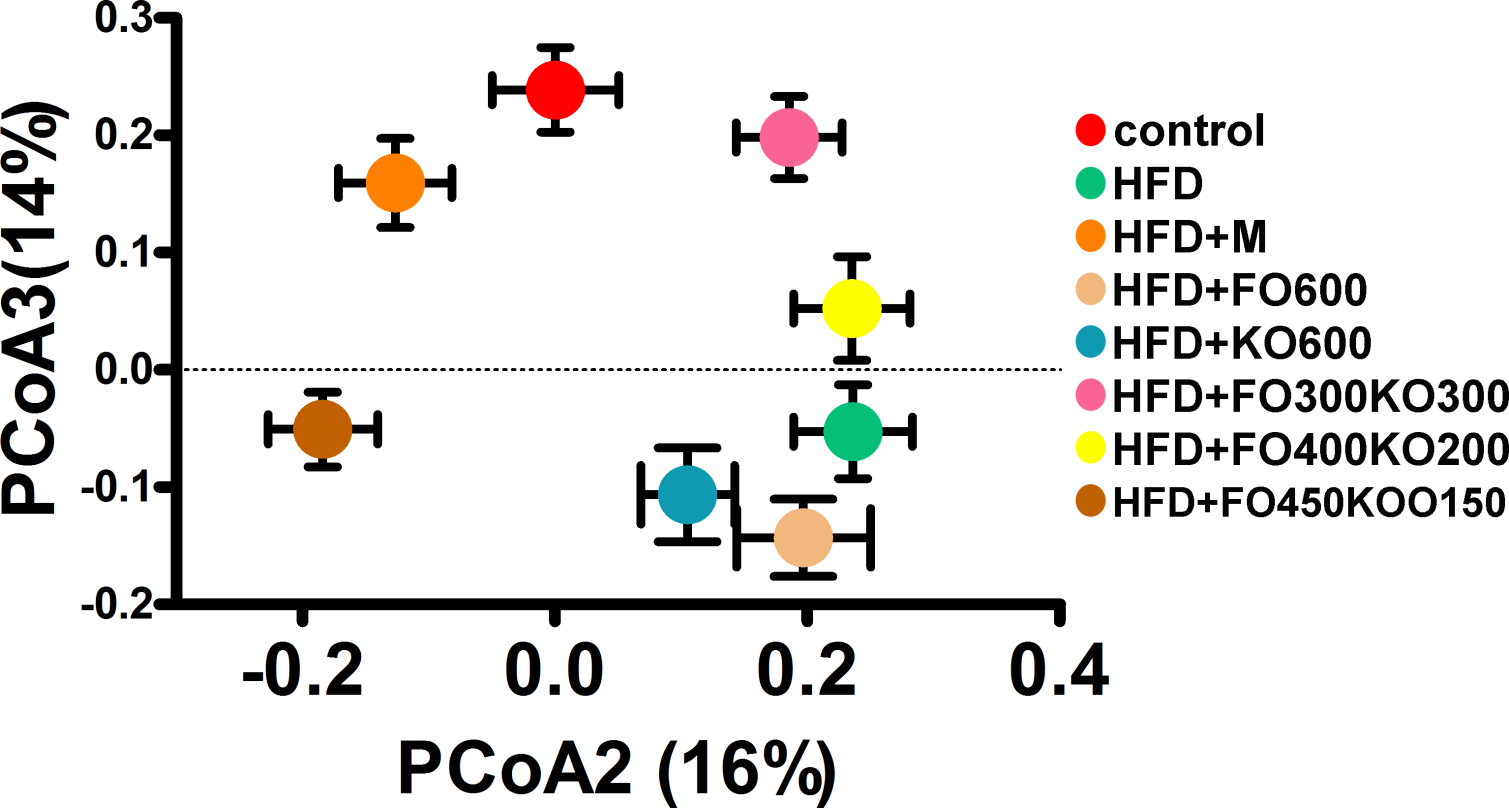
Change in the gut microbiota structure of mice fed the HFD and treated with different ratio of oil via Weighted Unifrac PCoA analysis. Data are presented as the means±S.D.

The overall compositions of the gut microbiota in the six groups were analyzed via PCoA (Fig 3, S1 Fig and S2 Fig). The mixtures of fish oil and krill oil shifted the overall composition of the intestinal microbiota in the HFD group towards that of the control, while the FO300KO300 group showed the closest relationship with the control, corresponding to the most significant effect on obesity alleviation. However, compared with the mixture oil treatment, the HFD+FO600 and HFD+KO600 groups showed relatively more similar structures with the HFD group. Interestingly, a close relationship was identified between the HFD+M and control groups in the structure of the gut microbiota, corresponding to significant reductions in LDL-C, TC and TG and attenuation of the features of hyperlipidemia in the HFD+M group.

### Microbial shifts based on taxon-based analysis

**Fig 4:**
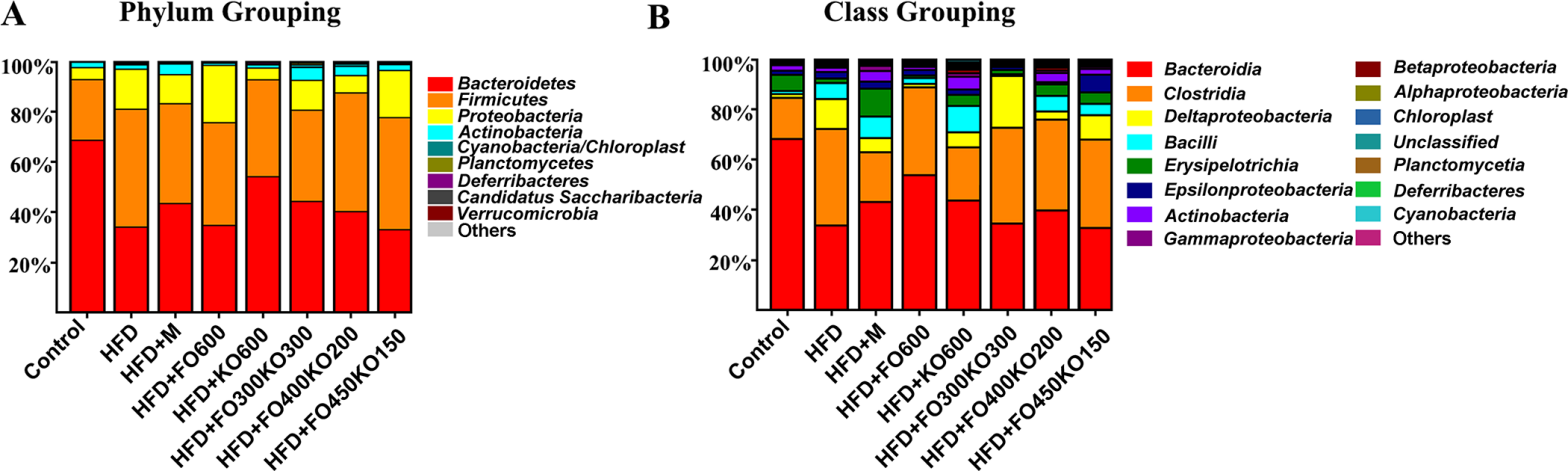
RDP classification of the sequences. (A) the phylum levels. (B) the class levels.

The RDP classifier was used to analyze the variation of intestinal microbial composition in animals receiving different oil supplements. The most abundant phyla included *Bacteroidetes*, *Firmicutes*, *Proteobacteria* and *Verrucomicrobia*. Two phyla, *Firmicutes* and *Bacteroidetes*, accounted for more than 77.45% of the total sequences in each group. The intestinal microorganism community structure had changed at the phylum level after 12 weeks of HFD feeding. Compared with the control group, the abundance of *Actinobacteria* (*P*>0.05) and *Bacteroidetes* (*P*<0.001) decreased, and the abundance of *Proteobacteria* (*P*<0.01) and *Firmicutes* (*P*<0.001) increased in the HFD group (S6 Table). The ratio of *Firmicutes* to *Bacteroidetes* was increased (*P*<0.05) after HFD treatment. Supplementation with oil decreased (*P*<0.05) this ratio compared with the HFD group. HFD+M and the other six experimental treatments mitigated the HFD-induced increase in *Firmicutes* (*P*<0.05 HFD+KO600, HFD+FO600 and HFD+FO300KO300 *vs* the HFD group; *P*>0.05 HFD+F0400KO200 and HFD+FO450KO150 *vs* the HFD group) and reduction in *Bacteroidete*s (Fig 4A and S6 Table, *P*<0.01 HFD+FO400KO200, HFD+KO600 and HFD+FO300KO300 *vs* the HFD group; *P*>0.05 HFD+F0600 and HFD+FO450KO150 *vs* the HFD group).

Within the phylum *Firmicutes*, *Bacilli* and *Clostridia* were the most common classes. The proportions of *Clostridia* (*P*<0.001) and *Bacilli* (*P*<0.01) increased after HFD treatment, while the ratio of *Bacteroidia* (*P*<0.001), the most abundant class of *Bacteroides*, reduced. The extra addition of krill oil, fish oil or their mixture partly restored the abundance of these three classes (Fig 4B and S7 Table).

Eight abundant genera (total DNA sequences with more than 1%) were identified in this study, of which four genera belonged to the phylum *Bacteroidetes*, two genera belonged to *Firmicutes* and two genera belonged to *Proteobacteria*. Among the 8 abundant genera, the frequencies of five genera increased after the HFD treatment (*P*<0.05), whereas the other three genera decreased (*P*<0.001). Furthermore, the different kinds of oil led to various effects on the genus abundance; detailed information is listed in S8 Table.

### Key phenotypes responding to the oil treatment in HFD fed mice

**Fig 5:**
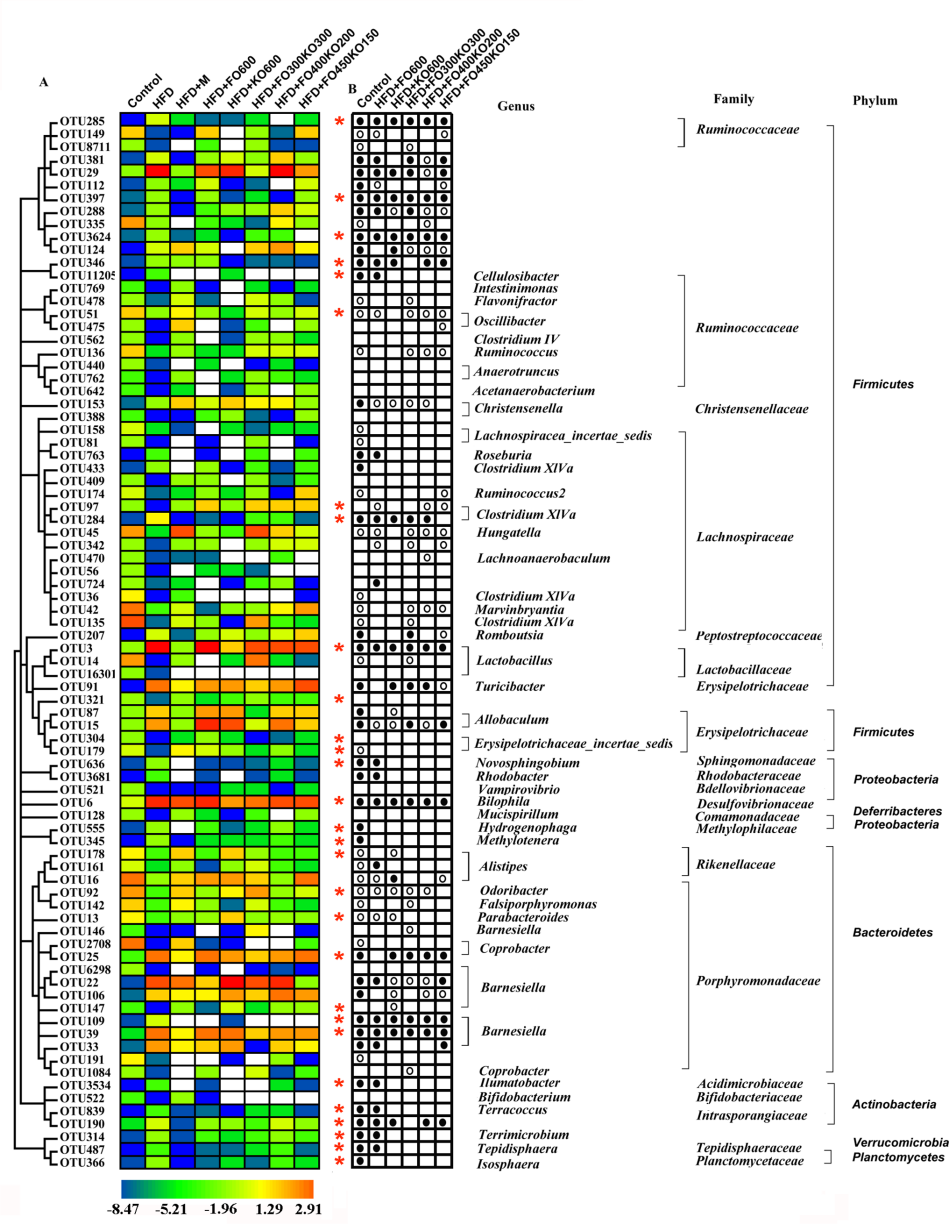
Eighty-two OTUs that were altered in abundance by an HFD and oil treatment based on redundancy analysis (RDA). (A) The color of the heatmap represents the normalized and log-transformed relative abundance of 82 OTUs. Undetected OTUs are represented in white. According to Mann-Whitney test, the rows correspond to 36 OTUs that were reduced and 46 OTUs that were enriched in HFD group compared with control group. (B) The directional changes of the 82 OTUs under the HFD and oil treatment. Dots represent reduced and circles represent enriched abundance of OTUs in the control group and oil groups compared with the HFD group. A description of the taxonomy (genus, family and phylum) of the OTUs is on the right. The red asterisks (*) represent the OTUs whose abundance was changed by HFD (*P*<0.05) and then restored by oil treatment (*P*<0.05).

**Fig 6:**
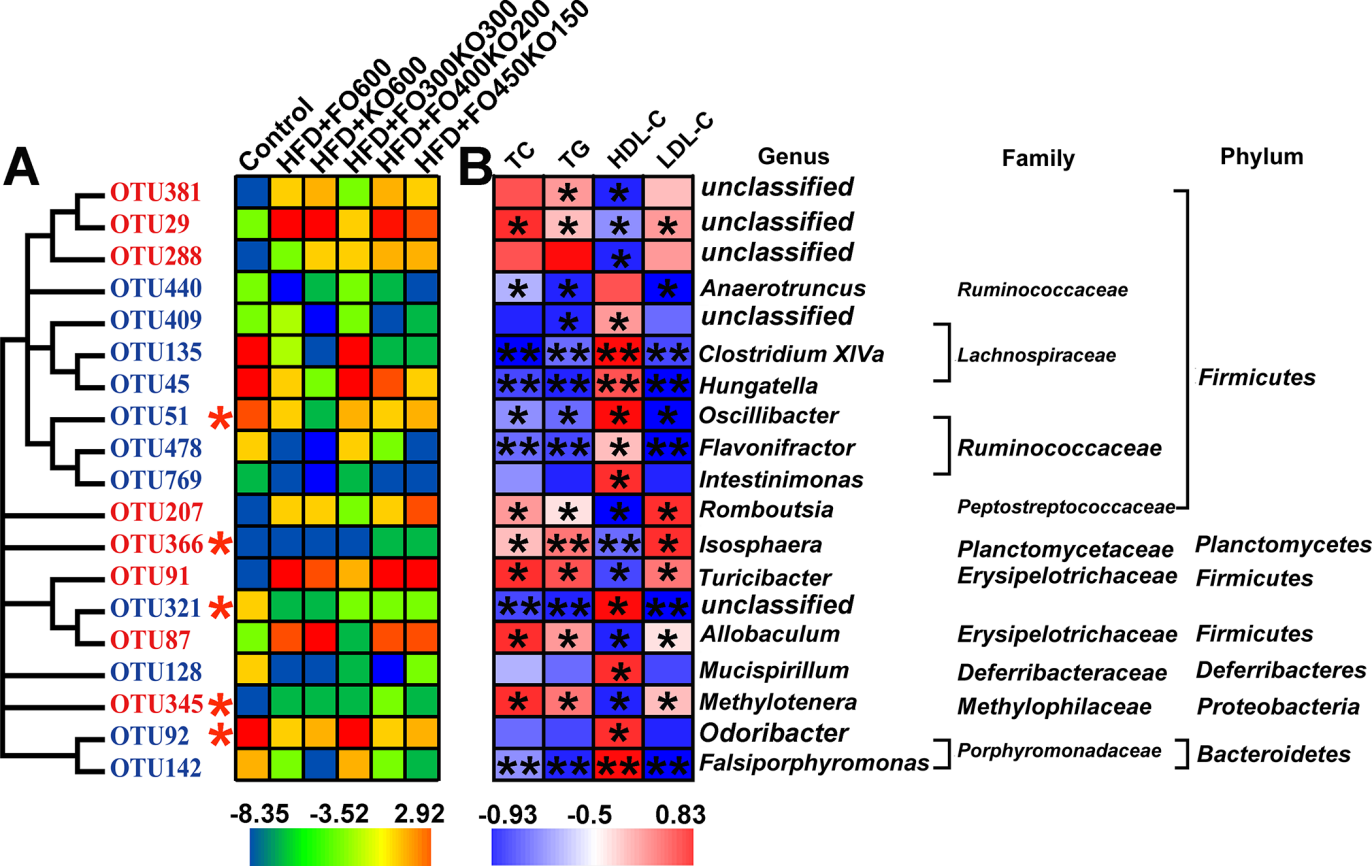
Nineteen oil-altered OTUs that were significantly correlated with host obesity parameters. (A) The color of the heatmap represents the normalized and log-transformed relative abundance of 19 OTUs. The rows corresponding to the OTUs are labeled on the left, blue represents the OTUs were negative correlated with obesity phenotype, and red represents the OTUs were positive correlated with obesity. The red asterisks (*) represents the OTUs whose abundance was changed by HFD and then restored by oil treatment (*P*<0.05). (B) The correlation between host obesity parameters and 19 OTUs. The degree of association between host parameters and OTU abundance, as computed by Spearman correlation analysis, is expressed by the intensity of colors. In red/blue squares, asterisks show that this correlation is significant, **P<0.01 and *P<0.05. The right shows the taxonomy of the OTUs.

Next, we wanted to identify specific intestinal bacteria. The gut microbiota might mediate the beneficial effects of oil supplementation on HFD-induced obesity. We used redundancy analysis models to appraise specially designated bacterial phylotypes. Oil treatment (HFD group vs each HFD+oil group) and HFD feeding (HFD group vs control group) changed the abundance of these specific bacterial phylotypes. Altogether, the abundance levels of 82 OTUs were changed by HFD feeding (S9 and S10 Tables). Supplementation with FO600, KO600, FO300KO300, FO400KO200 and FO450KO250 altered the abundance of 25, 36, 31, 28 and 31 OTUs, respectively (Fig 5 and S10 Table). Notably, oil supplementation altered the changes of 29 (out of 82) OTUs caused by an HFD (Fig 5 and S10 Table). Eleven OTUs were altered in all five oil treatment groups among the 82 OTUs changed by HFD feeding. Spearman’s correlation analysis was conducted between these 82 OTUs and specific plasma biochemical indicators. In total, 19 OTUs were significantly correlated with at least one plasma biochemical indicator. Eleven OTUs were negatively correlated with obesity disease phenotypes, because they were associated with decreased TC, TG and LDL-C and increased HDL-C (Fig 6), while eight of the OTUs were positively correlated with obesity phenotypes. The abundance levels of 5 OTUs were altered by all the oil groups compared with the HFD group (Figs 5 and 6).

Notably, three of the eleven OTUs negatively correlated with obesity phenotypes were increased by oil treatments (Fig 6). These three OTUs included bacteria belonging to *Hungatella* (*P*<0.05), *Oscillibacter* (*P*<0.05) *and Odoribacter* (*P*<0.01). Similarly, two of the eight OTUs that were positively correlated with obesity phenotypes were decreased by all oil treatment groups (Fig 6). These OTUs included *Isosphaera* (*P*<0.05) and *Methylotenera* (*P*<0.05).

## Discussion

This study examined the impact of dietary supplementation with fish oil, krill oil, or mixtures of the two on changes in body weight, adiposity index, liver index and gut microbiota in HFD-induced obese mice. A high-fat diet led to obesity in mice, with their body weight increasing 2.1-fold in 12 weeks (21.16±2.65 g at the starting point, 44.93±4.21 g at the endpoint). Dietary supplementation with FO300KO300 significantly reduced body weight gain, adiposity index and liver index. Just as we mentioned, restraining appetite is one of the mechanism for obesity management. In this study, the amount of food intake in different treatments had not been measured due to the actions like chew and molar generate a variable degree of food loss and waste, which make it difficult to accurately quantify the food intake. However, almost equal amounts of food surplus were observed in different treatments every day. On the other hand, previous studies found that dietary fish oil had no effect on appetite suppression [43], as well as the krill oil [44]. Appetite suppressed drugs might increase tolerance and dependency in a long-term clinical trials [11]. Therefore, we proposed that dietary supplementation of fish oil and/or krill oil is a much safer way to obesity alleviation.

In a previous study, Bashir et al suggested that fish oil, an oil rich in *n-3* PUFA, can be used as an insulin sensitizer in some reports and can reduce the inflammatory state of adipose tissue and decrease insulin resistance in obese mice [45]. The latent capacity of long-chain *n-3* PUFA against obesity-associated inflammation has been investigated in many studies [46]. Supplementation with DHA and EPA both revealed an anti-inflammatory effect with reduced high-sensitivity C-reactive protein, monocyte chemoattractant protein-1 and inflammatory mediators [47–50]. We observed that the body weight gain, liver index and adiposity index of the HFD+KO600 group were lower than those of the HFD+FO600 group. The content of EPA is higher in krill oil than in fish oil. EPA can increase the thromboxanes and 3-series prostaglandins, both of which play important anti-inflammatory roles [51]. However, in the HFD+FO300KO300 group, the ratio of EPA to DHA is closer to 1:1 compared with the other groups. Gabriel et al. proposed that, through lowering the production of the strong pro-inflammatory omega-6 arachidic acid, 2:1 and 1:1 EPA to DHA have more significant health benefits than 1:2 EPA to DHA [52].

Oil treatment was related to significant reductions in plasma TC, TG and HDL-C levels (Fig 2). The ratio of LDL-C to HDL-C is often used to assess the risk of coronary heart disease. In the HFD+FO300KO300 group, this ratio was significantly lower. Moreover, TC, TG and HDL-C levels in plasma were also significantly reduced by FO300KO300. These findings were consistent with a previous study of dietary krill oil and fish oil supplementation in high-fat-fed mice and overweight humans [53, 54]. A vast literature shows that fish oil and krill oil have potential benefits against chronic disorders such as cardiovascular diseases, endocannabinoid dysregulation, poor infant development, non-alcoholic fatty liver disease, premenstrual syndrome, inflammation and certain cancers [28, 55–57]. De Boer et al., suggest that *n-3* polyunsaturated fatty acids reduce inflammation, partly by increasing adiponectin and reducing the intensity of adipocyte-macrophage cross-linking, to reduce obesity related diseases [58]. Haider et al., suggested that the preventive effect of krill oil was attributable to the synergistic action among *n-3* PUFAs, phospholipids and astaxanthin [28]. However, several clinical studies reported that insulin resistance and glucose tolerance in patients with type 2 diabetes may be aggravated by high concentrations of *n-3* PUFAs [59]. Mixtures of the two oils contain a more balanced concentration of *n-3* polyunsaturated fatty acids (*n-3* PUFA), which might be the reason for the prominent effect of HFD+FO300KO300 in controlling obesity. These data show that the krill oil and fish oil mixtures are effective in controlling blood lipids, making them promising candidates for hyperlipidemia treatment.

The oil treatment also altered the structures of the gut microbiota in mice and eight abundant genera (include some acetate-producing bacteria) were identified in this study (S8 Table). Acetate had been reported to inhibit obesity-related inflammation and body fat accumulation or rodent diabetes through a variety of mechanisms [60, 61]. The majority of acetate-producing bacteria, such as *Barnesiella* [62], decreased (*P*<0.001) in the HFD group compared with the control and restored (*P*<0.01) after oil treatment in the HFD+FO300KO300 and HFD+FO400KO200 groups. Oil also has a significant effect on a number of well-known probiotics, such as *Lactobacillus* [63, 64]. HFD treatment significantly increased the ratio of *Lactobacillus* (*P*<0.001), and the subsequently oil treatment restored the abundance of this bacteria (S8 Table), which is in agreement with previous studies that *Lactobacillus* increased in obese patients [65]. It is interesting to find that the abundance of the *Helicobacter* was reduced after the oil treatment, which is known as a pathogenic bacteria for peptic ulcer disease [66].

Consistent with previous individual studies of fish oil, we found that supplementation with krill oil and fish oil apparently changed the gut microbiota of HFD-induced obese mice [67]. Changes in the composition of the gut microbiota can be caused by various environmental disturbances, whereas the same environmental stress factors may elicit different responses from different bacterial species in the same genus [68]. Thus, identifications of the changes in the species-level is very important. In this study, the abundance change profiles of 19 OTUs showed correlations with the obesity phenotype. Eight OTUs showed positive correlations with the obesity phenotype, including OTU381, OTU29, OTU288, OTU207, OTU366, OTU91, OTU87 and OTU345. Among them, *Allobaculum* (OTU87) is one of the short-chain fatty acid (SCFA)-producing bacteria in the gut. SCFA-producing bacteria can benefit the host through mitigating inflammation, protecting the mucosa from damage induced by pathogens and may contribute to obesity, insulin resistance and the alleviation of inflammation by reducing the intestinal endotoxins into the blood [69, 70]. On the other hand, 11 out of 19 OTUs showed negative correlations with the obesity phenotype, including OTU440, OTU409, OTU2135, OTU45, OTU51, OTU478, OTU769, OTU321, OTU128, OTU92 and OTU142. In addition, we found that *Oscillibacter* (OTU51, *P*<0.05), which is involved in the development of metabolic disorders or pro-inflammatory processes associated with obesity, enriched by almost all of the oil groups [71, 72]. For other key variables that were altered by oil treatment such as *Odoribacter* (OTU92, *P*<0.05 *vs* the HFD group), *Clostridium XlVa* (OTU135, *P*<0.05 vs HFD group), and *Flavonifractor* (OTU478, *P*<0.05, HFD+FO600, HGD+FO300KO300 and HFD+FO400KO200 group vs HFD group). *Odoribacter* was significantly increased in BALB/c mice in response to grid floor stress [73]. *Clostridium XlVa* is proved to affect various aspects of host biology, including intestinal epithelial barrier maintenance and food decomposition [74]. *Flavonifractor* has been found significantly enriched in fecal samples from non-obese compared with the obese subjects [75]. However, the mechanism whereby they may alleviate obesity remains unknown.

In conclusion, our studies showed that the treatment of fish oil, krill oil and their mixtures lead to the obesity alleviation, as well as the gut microbiota modulation. Fish oil and krill oil supplementation decreased the body weight gain, adiposity index and liver index, increased the abundance of the genera *Allobaculum*, *Odoribacter*, *Oscillibacter* and *Barnesiella* in the gut microbiota and decreased the proportion of *Lactobacillus*. Obesity development and alleviation showed differences in the gut microbiota in this study, and further studies are needed to address the causality between them.

## Supporting information

S1 Table The component of fatty acid in krill oil and fish oil. (PDF)

S2 Table The body weight of mice fed experimental diets. Data are represented as the means ± S.D and analyzed by ANOVA followed by Tukey post hoc test. *P<0.05, ** *P*<0.01 and ***P<0.001 *vs* the HFD group.
(PDF)

S3 Table Effect of supplementation with oil on plasma biochemical indicators of mice fed an HFD. Data are expressed in terms of mean ± S.D and analyzed by ANOVA followed by Tukey post hoc test. ***P<0.001, **P<0.01 and *P<0.05 all group compared with the Control group.
(PDF)

S4 Table Summary of sequence reads after quality control. (PDF)

S5 Table Microbial diversity of each group. Data are represented as the means ± S.D. and analyzed by Mann-Whitney test, **P<0.01 and *P<0.05 *vs* the HFD group.
(PDF)

S6 Table RDP classification of the sequence ratio at phylum level. Data are represented as the means ± S.D and analysed by Mann-Whitney test. ***P<0.001, **P<0.01 and *P<0.05 *vs* the HFD group.
(PDF)

S7 Table RDP classification of the sequence reads at class level. Data are presented as the means ± S.D and analyzed by Mann-Whitney test, ***P<0.001, **P<0.01 and *P<0.05 *vs* the HFD group.
(PDF)

S8 Table Eight abundant genera in all groups genus. Data are presented as the means ± S.D and analyzed by Mann-Whitney test, ***P<0.001, **P<0.01 and *P<0.05 *vs* the HFD group.
(PDF)

S9 Table Taxonomic assignments of 82 OTUs responding to oil treatment identified by redundancy analysis (RDA). (PDF)

S10 Table Relative abundance of the 82 OTUs responding to oil treatment identified by redundancy analysis (RDA). Data are presented as the means ± S.D and analyzed by Mann-Whitney test, ***P<0.001, **P<0.01 and *P<0.05 *vs* the HFD group.
(PDF)

S1 Fig Change in the gut microbiota structure of mice fed the HFD and treated with different ratio of oil via Unweighted Unifrac PCoA analysis. Data are presented as the means ± S.D. Each point represents the mean principal coordinate (PC) score of all mice in a group, and the error bar represents the S.D.
(TIF)

S2 Fig Change in the gut microbiota structure of mice fed the HFD and treated with different ratio of oil via Weighted Unifrac PCoA analysis. Data are presented as the means ± S.D. Each point represents the mean principal coordinate (PC) score of all mice in a group, and the error bar represents the S.D.
(TIF)
