# Supplementary material for: Modulation of the gut microbiota by the mixture of fish oil and krill oil in high-fat diet-induced obesity mice
Source: PLoS One. 2017 Oct 9;12(10):e0186216. doi: 10.1371/journal.pone.0186216 (PMC5633193; doi:10.1371/journal.pone.0186216)
Supplement: S1 Table — (PDF) [file pone.0186216.s001.pdf]

S1 Table. The component of fatty acid in krill oil and fish oil

		peak area(%)	
No.	compound	fish oil	krill oil
1	Dodecanoic acid	-	0.719288
2	Tetradecanoate	3.419262	26.70243
3	Pentadecanoic acid	1.294267	1.024069
4	Hexadecanoic acid	20.55666	-
5	Heptadecanoic acid	1.583855	-
6	Stearate	6.535653	2.098533
7	Eicosanoic acid	0.568188	-
8	11-Eicosenoic acid	1.061908	-
9	7-Hexadecenoic acid	0.803279	-
10	9-Hexadecenoic acid	4.670319	0.583936
11	9,12-Hexadecadienoic acid	-	0.837743
12	6-Hexadecenoic acid	1.906902	-
13	4,7,10,13-Hexadecatetraenoate	-	0.580976
14	6,9,12,15-Hexadecatetraenoate	-	2.394496
15	11-Octadecenoic acid	-	10.13226
16	9-Octadecenoic acid	14.07874	16.01075
17	Octadecanoic acid	2.207219	-
18	11,14-Octadecadienoic acid	1.385156	3.336659
19	9,12-Octadecadienoic acid	0.638078	4.083951
20	9,12,15-Octadecatrienoic acid	0.458493	-
21	Stearidonate	0.719468	9.771288
22	10,13-Eicosadienoic acid,	0.985108	-
23	5,8,11,14-Eicosatetraenoic acid	2.256837	-
24	8,11,14,17-Eicosatetraenoate	-	-
25	EPA	5.673304	17.81914
26	13-Docosenoic acid	-	-
27	4,7,10,13,16-Docosapentaenoate	2.681636	-
28	7,10,13,16,19-Docosapentaenoate	0.758215	-
29	DHA	25.75744	3.904475
