# Supplementary material for: Modulation of the gut microbiota by the mixture of fish oil and krill oil in high-fat diet-induced obesity mice
Source: PLoS One. 2017 Oct 9;12(10):e0186216. doi: 10.1371/journal.pone.0186216 (PMC5633193; doi:10.1371/journal.pone.0186216)
Supplement: S2 Table — Data are represented as the means ± S.D and analyzed by ANOVA followed by Tukey post hoc test. *P<0.05, ** P<0.01 and ***P<0.001 vs the HFD group. (PDF) [file pone.0186216.s002.pdf]

Table S2. **The body weight of mice fed experimental diets.** Data are presented as the means ± S.D and analyzed by ANOVA followed by Tukey post hoc test. *P<0.05, ** P<0.01 and ***P<0.001 *vs* the HFD group.

	Normal diet	High -fat diet						
	Control	HFD	HFD+M	HFD+FO600	HFD+KO600	FO300KO300	FO400KO200	FO450KO150
Initial BW (g)	23.33±3.23	22.16±2.65	23.97±3.13	23.44±2.46	23.26±3.17	24.12±3.43	23.66±2.01	24.01±3.19
Final BW(g)	32.14±3.72	44.93±4.21	37.86±4.01	41.63±3.17	40.13±4.36	38.77±4.21	41.26±4.33	43.17±4.13
BW gain (g)	9.47±1.33***	22.66±1.67	13.44±2.03***	17.62±1.56**	16.27±1.78**	14.61±2.21**	17.81±1.88**	19.32±1.32*
