# Supplementary material for: Modulation of the gut microbiota by the mixture of fish oil and krill oil in high-fat diet-induced obesity mice
Source: PLoS One. 2017 Oct 9;12(10):e0186216. doi: 10.1371/journal.pone.0186216 (PMC5633193; doi:10.1371/journal.pone.0186216)
Supplement: S3 Table — Data are expressed in terms of mean ± S.D and analyzed by ANOVA followed by Tukey post hoc test. ***P<0.001, **P<0.01 and *P<0.05 all group compared with the Control group. (PDF) [file pone.0186216.s003.pdf]

Table S3. Effect of supplementation with oil on plasma biochemical indicators of mice fed an HFD. Data are represented as the means ± S.D and analyzed by ANOVA followed by Tukey post hoc test. ***P<0.001, **P<0.01 and *P<0.05 all group compared with the Control group.

	Control	HFD	HFD+M	HFD+FO600	HFD+KO600	HFD+FO300KO300	HFD+FO400KO200	HFD+FO450KO150
TC	3.72 ± 0.56	5.02 ± 1.14***	4.37 ± 0.77***	4.73 ± 1.21*	4.45 ± 1.01*	4.4 ± 1.17**	4.43 ± 0.98**	4.59 ± 1.45
TG	0.75 ± 0.12	1.57 ± 0.54**	0.99 ± 0.14***	1.39 ± 0.73	1.28 ± 0.68*	1.21 ± 0.76***	1.28 ± 0.25*	1.29 ± 0.39*
HDL-C	5.19 ± 1.69	4.07 ± 1.07***	4.96 ± 1.21***	4.51 ± 1.14**	4.85 ± 1.22**	4.88 ± 1.24**	4.79 ± 1.31*	4.78 ± 1.33
LDL-C	1.01 ± 0.47	2.17 ± 0.76***	1.25 ± 0.63***	1.98 ± 0.55*	1.43 ± 0.73***	1.37 ± 0.62***	1.41 ± 0.54***	1.47 ± 0.69***
