# Supplementary material for: Modulation of the gut microbiota by the mixture of fish oil and krill oil in high-fat diet-induced obesity mice
Source: PLoS One. 2017 Oct 9;12(10):e0186216. doi: 10.1371/journal.pone.0186216 (PMC5633193; doi:10.1371/journal.pone.0186216)
Supplement: S4 Table — (PDF) [file pone.0186216.s004.pdf]

Table S4. Summary of sequence reads after quality control

Group	Raw num	Clean num	Out target num	Chimeras num	Filtered num
Control	35233	34330	616	2479	31235
HFD	32397	31717	869	1771	29077
HFD+M	34571	33881	1453	1164	31264
HFD+FO600	32265	31213	1144	2034	28035
HFD+KO600	40649	39699	863	3176	35660
HFD+FO300KO300	37239	36384	820	3029	32535
HFD+FO400KO200	37272	36491	1670	1301	33520
HFD+FO450KO150	41951	41081	1872	2775	36434
