# Supplementary material for: Modulation of the gut microbiota by the mixture of fish oil and krill oil in high-fat diet-induced obesity mice
Source: PLoS One. 2017 Oct 9;12(10):e0186216. doi: 10.1371/journal.pone.0186216 (PMC5633193; doi:10.1371/journal.pone.0186216)
Supplement: S5 Table — Data are represented as the means ± S.D. and analyzed by Mann-Whitney test, **P<0.01 and *P<0.05 vs the HFD group. (PDF) [file pone.0186216.s005.pdf]

S5 Table. Microbial diversity of each group. Data are represented as the means ± S.D. and analyzed by Mann-Whitney test, ***P*<0.01 and **P*<0.05 *vs* the HFD group.

Groups	Shannon index	ACE index	Chao1 index	Simpson	Coverage
Control	5.20±0.43	2730.77±524	2108.47±432	0.01±0.0024	0.98
HFD	4.59±0.74*	2702.35±423	1920.25±324	0.03±0.0015*	0.98
HFD+M	4.89±1.07*	2449.08±376	1777.53±354*	0.02±0.0031	0.98
HFD+FO600	4.78±1.21	1935.63±234**	1472.04±315*	0.02±0.0019	0.99
HFD+KO600	4.86±0.88	2743.34±643	2122.68±327	0.02±0.0043	0.98
HFD+FO300KO300	5.1±0.97	2776.64±387	2216.93±369	0.02±0.0052	0.98
HFD+FO400KO200	4.86±1.01	2907.67±424*	2111.37±433	0.02±0.0033	0.98
HFD+FO450KO150	4.75±1.24*	3017.46±412*	2346.54±451	0.03±0.0065	0.98
