# Supplementary material for: Modulation of the gut microbiota by the mixture of fish oil and krill oil in high-fat diet-induced obesity mice
Source: PLoS One. 2017 Oct 9;12(10):e0186216. doi: 10.1371/journal.pone.0186216 (PMC5633193; doi:10.1371/journal.pone.0186216)
Supplement: S6 Table — Data are represented as the means ± S.D and analysed by Mann-Whitney test. ***P<0.001, **P<0.01 and *P<0.05 vs the HFD group. (PDF) [file pone.0186216.s006.pdf]

Table S6. RDP classification of the sequence ratio at phylum level. Data are represented as the means ± S.D and analysed by Mann-Whitney test. ****P*<0.001, ***P*<0.01 and **P*<0.05 *vs* the HFD group.

Phylum	Control (%)	HFD (%)	HFD+M (%)	HFD+FO600 (%)	HFD+KO600 (%)	HFD+ FO300KO300 (%)	HFD+ FO400KO200 (%)	HFD+ FO450KO150 (%)
Bacteroidetes	68.44±4.67↑***	33.88±3.21	43.3±4.22↑***	34.58±4.21↑	53.96±4.31↑***	44.11±5.42↑***	40.06±5.17↑**	32.87±4.88↓
Firmicutes	24.13±2.42↓***	46.93±3.34	39.68±3.17↓**	40.87±3.47↓**	38.55±8.75↓**	36.32±3.41↓**	47.23±3.43↑	44.58±5.87↓
Proteobacteria	4.76±1.22↓**	15.89±2.33	11.56±2.19↓*	22.83±2.21↑**	4.67±1.15↓**	11.87±2.45↓**	6.9±2.13↓**	18.76±2.16↑*
Actinobacteria	2.17±0.89↑	1.74±0.67	4.29±1.78↑*	0.79±0.11↓**	1.25±0.32↓	5.1±1.88↑*	3.65±↑*	2.32±0.88↑
Cyanobacteria/Chloroplast	0.15±0.04↓	0.3±0.02	0.36±0.13↑	0.1±0.012↓	0.21±0.05↓	1.09±0.21↑*	0.98±0.12↑*	0.48±0.16↑
Planctomycetes	0.12±0.02↓*	0.22±0.11	0.33±0.10↑	0.08±0.001↓*	0.16±0.07↓	0.68±0.04↑*	0.54±0.16↑*	0.22±0.09
Deferribacteres	0.01±0.001↓*	0.28±0.12	0.05±0.01↓**	0.45±0.06↑*	0.61±0.11↑*	0.18±0L08↓	0.02±0.01↓	0.41±0.07↑*
Candidatus Saccharibacteria	0.05±0.01↓**	0.46±0.11	0.15±0.02↓**	0.15±0.02↓*	0.34±0.08↓	0.05±0.01↓*	0.04±0.01↓*	0.07±0.01↓*
Verrucomicrobia	0.07±0.02↓	0.15±0.03	0.14±0.01↓	0.04±0.001↓*	0.06±0.01↓*	0.38±0.10↑*	0.29±0.07↑*	0.14±0.03↓
Unclassified	0.05±0.01↓	0.07±0.01	0.07±0.02↓	0.08±0.023↑	0.11±0.02↑	0.04±0.01↓	0.13±0.04↑	0.08±0.01↑
