# Supplementary material for: Modulation of the gut microbiota by the mixture of fish oil and krill oil in high-fat diet-induced obesity mice
Source: PLoS One. 2017 Oct 9;12(10):e0186216. doi: 10.1371/journal.pone.0186216 (PMC5633193; doi:10.1371/journal.pone.0186216)
Supplement: S7 Table — Data are presented as the means ± S.D and analyzed by Mann-Whitney test, ***P<0.001, **P<0.01 and *P<0.05 vs the HFD group. (PDF) [file pone.0186216.s007.pdf]

S7 Table. RDP classification of the sequence reads at class level. Data are presented as the means ± S.D and analyzed by Mann-Whitney test, ****P*<0.001, ***P*<0.01 and **P*<0.05 *vs* the HFD group.

Class	Control (%)	HFD (%)	HFD+M (%)	HFD+FO600 (%)	HFD+KO600 (%)	HFD+FO300KO300 (%)	HFD+FO400KO200 (%)	HFD+FO450KO150 (%)
Bacteroidia	68.38±6.41↑***	33.77±4.88	43.16±8.74↑*	53.9±10.37↑**	43.73±6.89↑**	34.53±6.88↑	39.78±8.89↑	32.75±5.67↓
Clostridia	16.46±3.16↓***	38.63±6.74	19.91±4.32↓***	35.14±7.62↓	21.29±3.41↓***	38.34±5.96↓	36.34±7.42↓	35.4±5.87↓
Deltaproteobacteria	1.57±0.38↓***	11.95±2.65	5.67±1.32↓**	1.35±0.23↓***	6.01±2.46↓**	20.73±4.67↑**	3.26±2.34↓***	9.74±4.67↓
Bacilli	1.17±0.24↓**	6.42±1.17	8.63±2.13↑	2.38±0.34↓***	10.57±3.11↑*	0.69±0.12↓***	6.23±1.47↓	4.57±1.37↓
Erysipelotrichia	6.45±1.13↑**	1.81±0.44	11.13±2.24↑***	1.01±0.18↓	4.44±1.56↑**	1.71±0.22↓	4.65±1.33↑**	4.56±1.42↑**
Epsilonproteobacteria	1.69±0.32↓*	2.59±0.32	2.8±0.43↑	2.21±0.28↓	2.15±0.98↓	1.19±0.07↓*	0.87±0.16↓***	7.06±2.17↑***
Actinobacteria	2.17±0.47↑*	1.74±0.27	4.29±0.26↑	1.25±0.16↓	5.1±1.17↑**	0.79±0.04↓*	3.65±0.78↑***	2.32±0.43↑**
Gammaproteobacteria	0.52±011↑	0.47±0.13	2.06±0.17↑**	0.48±0.24↑	1.36±0.05↑***	0.56±0.26↑***	0.96±0.22↑***	0.9±0.26↑***
Betaproteobacteria	0.8±0.16↑	0.59±0.24	0.77±0.06↑	0.43±0.17↓	1.5±0.17↑**	0.21±0.01↓***	1.2±0.32↑***	0.7±0.12↑**
Alphaproteobacteria	0.18±0.03↓	0.28±0.04	0.25±0.05↓	0.21±0.09↓	0.84±0.11↑*	0.13±0.04↓*	0.59±0.18↑***	0.36±0.11↑**
Chloroplast	0.11±0.04*↓	0.21±0.05	0.26±0.11↑	0.16±0.05↓	0.79±0.23↑**	0.07±0.01↓**	0.73±0.25↑***	0.36±0.14↑
Unclassified	0.15±0.01↓*	0.59±0.03	0.25±0.03↓	0.5±0.07↓	0.17±0.06↓*	0.38±0.02↓	0.25±0.11↓	0.2±0.07↓**
Planctomycetia	0.09±0.01↓*	0.18±0.07	0.29±0.09↑	0.14±0.03↓	0.59±0.08↑**	0.06±0.01↓*	0.47±0.14↑***	0.2±0.09↑**
Deferribacteres	0.01±0.002↓**	0.28±0.03	0.05±0.001↓*	0.61±0.17↑**	0.18±0.04↓	0.45±0.02↑***	0.02±0.005↓***	0.41±0.03↑***
Cyanobacteria	0.04±0.013↓	0.09±0.01	0.1±0.01↑	0.05±0.01↓	0.31±0.13↑**	0.03±0.006↓	0.25±0.02↑	0.11±0.04↑
Spartobacteria	0.03±0.005↓	0.07±0.01	0.07±0.01	0.04±0.003↓	0.21±0.09↑	0.02±0.003↓	0.13±0.04↑	0.07±0.01↓
Sphingobacteriia	0.02±0.002↓	0.04±0.001	0.09±0.02↑	0.03±0.005↓	0.18±0.04↑*	0.02±0.004↓*	0.15±0.03↑***	0.07±0.02↑**
Flavobacteriia	0.03±0.004↓	0.06±0.002	0.03±0.001↓	0.03±0.001↓	0.13±0.01↑**	0.03±0.004↓*	0.09±0.01↑*	0.03±0.006*
Phycisphaerae	0.03±0.005↓	0.04±0.001	0.04±0.002	0.02±0.001↓	0.09±0.02↑	0.02±0.002↓	0.08±0.02↑	0.02±0.002↓
Opitutae	0.02±0.003↓	0.03±0.003	0.02±0.001↓	0.01±0.001↓	0.07±0.01↑	0.04±0.006↓	0.09±0.01↑	0.03±0.004↑
