# Supplementary material for: Modulation of the gut microbiota by the mixture of fish oil and krill oil in high-fat diet-induced obesity mice
Source: PLoS One. 2017 Oct 9;12(10):e0186216. doi: 10.1371/journal.pone.0186216 (PMC5633193; doi:10.1371/journal.pone.0186216)
Supplement: S8 Table — Data are presented as the means ± S.D and analyzed by Mann-Whitney test, ***P<0.001, **P<0.01 and *P<0.05 vs the HFD group. (PDF) [file pone.0186216.s008.pdf]

S8 Table. Eight abundant genera in all groups genus. Data are presented as the means ± S.D and analyzed by Mann-Whitney test, ***P<0.001, **P<0.01 and *P<0.05 *vs* the HFD group.

Phylum	Genus	Control (%)	HFD (%)	HFD+M (%)	HDHF+FO600 (%)	HFD+KO600 (%)	HFD+FO300KO300 (%)	HFD+FO400KO200 (%)	HFD+FO450KO150 (%)
Bacteroidetes	*Barnesiella*	38.02±4.63↑***	26.33±2.13	29.87±3.26*↑	17.05±2.54**↓	21.36±4.23*↓	34.78±3.87**↑	31.91±3.63**↑	19.98±4.83**↓
Proteobacteria	*Bilophila*	1.4±0.74↓***	4.67±1.11	5.32±1.37↑	10.82±3.63***↑	10.56±2.71***↑	1.26±0.53***↓	2.67±1.13**↓	6.71±1.21**↑
Firmicutes	*Lactobacillus*	0.81±0.03↓***	8.00±2.11	9.69±5.41*↑	1.42±0.25***↓	6.19±1.77*↓	1.08±0.42***↓	5.56±1.20*↓	3.75±1.10*↓
Firmicutes	*Clostridium XlVa*	0.88±0.14↓***	1.97±0.86	1.63±0.32↓	3.66±1.14**↑	5.68±1.86***↑	10.8±3.16***↓	4.41±1.29**↑	5.29±1.21***↑
Bacteroidetes	*Tannerella*	3.9±1.11↑***	2.65±0.27	2.93±1.19*↑	1.93±0.36**↓	2.13±0.29*↓	1.97±0.73*↓	3.64±1.20***↑	2.75±1.34↓
Proteobacteria	*Helicobacter*	1.68±0.21↓*	2.79±0.77	2.12±0.77↓	1.19±0.43*↓	2.59±1.10↓	2.21±0.62↓	1.85±0.29*↓	7.05±2.10↑
Bacteroidetes	*Coprobacter*	3.03±0.43↓***	3.8±0.17	2.68±0.21***↓	1.92±0.28***↓	1.58±0.75***↓	2.71±1.17↓	2.34±0.39**↓	2.92±0.98↓
Bacteroidetes	*Bacteroides*	7.33±1.76↑***	2.14±0.54	1.57±0.21*↓	2.64±0.29↑	1.04±0.87*↓	2.65±1.09↑	1.91±0.76↓	1.04±0.66**↓
