# Supplementary material for: Modulation of the gut microbiota by the mixture of fish oil and krill oil in high-fat diet-induced obesity mice
Source: PLoS One. 2017 Oct 9;12(10):e0186216. doi: 10.1371/journal.pone.0186216 (PMC5633193; doi:10.1371/journal.pone.0186216)
Supplement: S9 Table — (PDF) [file pone.0186216.s009.pdf]

Table S9. Taxonomic assignments of 82 OTUs responding to oil treatment identified by redundancy analysis (RDA).

	phylum	class	order	family	genus
OTU2708	*Bacteroidetes*	*Bacteroidia*	*Bacteroidales*	*Porphyromonadaceae*	*Coprobacter*
OTU14	*Firmicutes*	*Bacilli*	*Lactobacillales*	*Lactobacillaceae*	*Lactobacillus*
OTU135	*Firmicutes*	*Clostridia*	*Clostridiales*	*Lachnospiraceae*	*Clostridium XlVa*
OTU149	*Firmicutes*	*Clostridia*	*Clostridiales*	*Ruminococcaceae*	*Unclassified*
OTU36	*Firmicutes*	*Clostridia*	*Clostridiales*	*Lachnospiraceae*	*Clostridium XlVa*
OTU42	*Firmicutes*	*Clostridia*	*Clostridiales*	*Lachnospiraceae*	*Marvinbryantia*
OTU45	*Firmicutes*	*Clostridia*	*Clostridiales*	*Lachnospiraceae*	*Hungatella*
OTU136	*Firmicutes*	*Clostridia*	*Clostridiales*	*Ruminococcaceae*	*Ruminococcus*
OTU191	*Bacteroidetes*	*Bacteroidia*	*Bacteroidales*	*Porphyromonadaceae*	*Barnesiella*
OTU81	*Firmicutes*	*Clostridia*	*Clostridiales*	*Lachnospiraceae*	*Lachnospiracea_incertae_sedis*
OTU56	*Firmicutes*	*Clostridia*	*Clostridiales*	*Lachnospiraceae*	*Unclassified*
OTU128	*Deferribacteres*	*Deferribacteres*	*Deferribacterales*	*Deferribacteraceae*	*Mucispirillum*
OTU6298	*Bacteroidetes*	*Bacteroidia*	*Bacteroidales*	*Porphyromonadaceae*	*Barnesiella*
OTU475	*Firmicutes*	*Clostridia*	*Clostridiales*	*Ruminococcaceae*	*Oscillibacter*
OTU92	*Bacteroidetes*	*Bacteroidia*	*Bacteroidales*	*Porphyromonadaceae*	*Odoribacter*
OTU97	*Firmicutes*	*Clostridia*	*Clostridiales*	*Lachnospiraceae*	*Clostridium XlVa*
OTU179	*Firmicutes*	*Erysipelotrichia*	*Erysipelotrichales*	*Erysipelotrichaceae*	*Erysipelotrichaceae_incertae_sedis*
OTU304	*Firmicutes*	*Erysipelotrichia*	*Erysipelotrichales*	*Erysipelotrichaceae*	*Erysipelotrichaceae_incertae_sedis*
OTU8711	*Firmicutes*	*Clostridia*	*Clostridiales*	*Ruminococcaceae*	*Unclassified*
OTU562	*Firmicutes*	*Clostridia*	*Clostridiales*	*Ruminococcaceae*	*Clostridium IV*
OTU335	*Firmicutes*	*Clostridia*	*Clostridiales*	*Unclassified*	*Unclassified*
OTU409	*Firmicutes*	*Clostridia*	*Clostridiales*	*Lachnospiraceae*	*Unclassified*

OTU521	*Proteobacteria*	*Deltaproteobacteria*	*Bdellovibrionales*	*Bdellovibrionaceae*	*Vampirovibrio*
OTU342	*Firmicutes*	*Clostridia*	*Clostridiales*	*Lachnospiraceae*	*Unclassified*
OTU51	*Firmicutes*	*Clostridia*	*Clostridiales*	*Ruminococcaceae*	*Oscillibacter*
OTU174	*Firmicutes*	*Clostridia*	*Clostridiales*	*Lachnospiraceae*	*Ruminococcus2*
OTU142	*Bacteroidetes*	*Bacteroidia*	*Bacteroidales*	*Porphyromonadaceae*	*Falsiporphyromonas*
OTU1084	*Bacteroidetes*	*Bacteroidia*	*Bacteroidales*	*Porphyromonadaceae*	*Coprobacter*
OTU440	*Firmicutes*	*Clostridia*	*Clostridiales*	*Ruminococcaceae*	*Anaerotruncus*
OTU13	*Bacteroidetes*	*Bacteroidia*	*Bacteroidales*	*Porphyromonadaceae*	*Parabacteroides*
OTU161	*Bacteroidetes*	*Bacteroidia*	*Bacteroidales*	*Rikenellaceae*	*Alistipes*
OTU762	*Firmicutes*	*Clostridia*	*Clostridiales*	*Ruminococcaceae*	*Anaerotruncus*
OTU178	*Bacteroidetes*	*Bacteroidia*	*Bacteroidales*	*Rikenellaceae*	*Alistipes*
OTU146	*Bacteroidetes*	*Bacteroidia*	*Bacteroidales*	*Porphyromonadaceae*	*Barnesiella*
OTU147	*Bacteroidetes*	*Bacteroidia*	*Bacteroidales*	*Porphyromonadaceae*	*Barnesiella*
OTU388	*Firmicutes*	*Clostridia*	*Clostridiales*	*Lachnospiraceae*	*Unclassified*
OTU642	*Firmicutes*	*Clostridia*	*Clostridiales*	*Ruminococcaceae*	*Acetanaerobacterium*
OTU16	*Bacteroidetes*	*Bacteroidia*	*Bacteroidales*	*Rikenellaceae*	*Alistipes*
OTU470	*Firmicutes*	*Clostridia*	*Clostridiales*	*Lachnospiraceae*	*Lachnoanaerobaculum*
OTU16301	*Firmicutes*	*Bacilli*	*Lactobacillales*	*Lactobacillaceae*	*Lactobacillus*
OTU158	*Firmicutes*	*Clostridia*	*Clostridiales*	*Lachnospiraceae*	*Lachnospiracea_incertae_sedis*
OTU478	*Firmicutes*	*Clostridia*	*Clostridiales*	*Ruminococcaceae*	*Flavonifractor*
OTU724	*Firmicutes*	*Clostridia*	*Clostridiales*	*Lachnospiraceae*	*Unclassified*
OTU321	*Unclassified*	*Unclassified*	*Unclassified*	*Unclassified*	*Unclassified*
OTU522	*Actinobacteria*	*Actinobacteria*	*Bifidobacteriales*	*Bifidobacteriaceae*	*Bifidobacterium*
OTU769	*Firmicutes*	*Clostridia*	*Clostridiales*	*Ruminococcaceae*	*Intestinimonas*
OTU112	*Firmicutes*	*Clostridia*	*Clostridiales*	*NA*	*Unclassified*

OTU636	*Proteobacteria*	*Alphaproteobacteria*	*Sphingomonadales*	*Sphingomonadaceae*	*Novosphingobium*
OTU839	*Actinobacteria*	*Actinobacteria*	*Actinomycetales*	*Intrasporangiaceae*	*Terracoccus*
OTU3534	*Actinobacteria*	*Actinobacteria*	*Acidimicrobiales*	*Acidimicrobiaceae*	*Ilumatobacter*
OTU397	*Firmicutes*	*Clostridia*	*Clostridiales*	*NA*	*Unclassified*
OTU366	*Planctomycetes*	*Planctomycetia*	*Planctomycetales*	*Planctomycetaceae*	*Isosphaera*
OTU3624	*Firmicutes*	*Clostridia*	*Clostridiales*	*Unclassified*	*Unclassified*
OTU190	*Actinobacteria*	*Actinobacteria*	*Actinomycetales*	*Unclassified*	*Unclassified*
OTU153	*Firmicutes*	*Clostridia*	*Clostridiales*	*Christensenellaceae*	*Christensenella*
OTU3681	*Proteobacteria*	*Alphaproteobacteria*	*Rhodobacterales*	*Rhodobacteraceae*	*Rhodobacter*
OTU288	*Firmicutes*	*Clostridia*	*Clostridiales*	*Unclassified*	*Unclassified*
OTU433	*Firmicutes*	*Clostridia*	*Clostridiales*	*Lachnospiraceae*	*Clostridium XlVa*
OTU487	*Planctomycetes*	*Phycisphaerae*	*Tepidisphaerales*	*Tepidisphaeraceae*	*Tepidisphaera*
OTU763	*Firmicutes*	*Clostridia*	*Clostridiales*	*Lachnospiraceae*	*Roseburia*
OTU87	*Firmicutes*	*Erysipelotrichia*	*Erysipelotrichales*	*Erysipelotrichaceae*	*Allobaculum*
OTU555	*Proteobacteria*	*Betaproteobacteria*	*Burkholderiales*	*Comamonadaceae*	*Hydrogenophaga*
OTU11205	*Firmicutes*	*Clostridia*	*Clostridiales*	*Ruminococcaceae*	*Cellulosibacter*
OTU314	*Verrucomicrobia*	*Spartobacteria*	*NA*	*NA*	*Terrimicrobium*
OTU346	*Firmicutes*	*Clostridia*	*Clostridiales*	*Unclassified*	*Unclassified*
OTU15	*Firmicutes*	*Erysipelotrichia*	*Erysipelotrichales*	*Erysipelotrichaceae*	*Allobaculum*
OTU109	*Bacteroidetes*	*Bacteroidia*	*Bacteroidales*	*Porphyromonadaceae*	*Unclassified*
OTU6	*Proteobacteria*	*Deltaproteobacteria*	*Desulfovibrionales*	*Desulfovibrionaceae*	*Bilophila*
OTU345	*Proteobacteria*	*Betaproteobacteria*	*Methylophilales*	*Methylophilaceae*	*Methylotenera*
OTU381	*Firmicutes*	*Clostridia*	*Clostridiales*	*Unclassified*	*Unclassified*
OTU106	*Bacteroidetes*	*Bacteroidia*	*Bacteroidales*	*Porphyromonadaceae*	*Barnesiella*
OTU25	*Bacteroidetes*	*Bacteroidia*	*Bacteroidales*	*Porphyromonadaceae*	*Coprobacter*

OTU284	*Firmicutes*	*Clostridia*	*Clostridiales*	*Lachnospiraceae*	*Clostridium XlVa*
OTU124	*Firmicutes*	*Clostridia*	*Clostridiales*	*Unclassified*	*Unclassified*
OTU207	*Firmicutes*	*Clostridia*	*Clostridiales*	*Peptostreptococcaceae*	*Romboutsia*
OTU3	*Firmicutes*	*Bacilli*	*Lactobacillales*	*Lactobacillaceae*	*Lactobacillus*
OTU285	*Firmicutes*	*Clostridia*	*Clostridiales*	*Ruminococcaceae*	*Unclassified*
OTU33	*Bacteroidetes*	*Bacteroidia*	*Bacteroidales*	*Porphyromonadaceae*	*Barnesiella*
OTU39	*Bacteroidetes*	*Bacteroidia*	*Bacteroidales*	*Porphyromonadaceae*	*Barnesiella*
OTU29	*Firmicutes*	*Clostridia*	*Clostridiales*	*Unclassified*	*Unclassified*
OTU91	*Firmicutes*	*Erysipelotrichia*	*Erysipelotrichales*	*Erysipelotrichaceae*	*Turicibacter*
OTU22	*Bacteroidetes*	*Bacteroidia*	*Bacteroidales*	*Porphyromonadaceae*	*Barnesiella*
