# Supplementary material for: Modulation of the gut microbiota by the mixture of fish oil and krill oil in high-fat diet-induced obesity mice
Source: PLoS One. 2017 Oct 9;12(10):e0186216. doi: 10.1371/journal.pone.0186216 (PMC5633193; doi:10.1371/journal.pone.0186216)
Supplement: S10 Table — Data are presented as the means ± S.D and analyzed by Mann-Whitney test, ***P<0.001, **P<0.01 and *P<0.05 vs the HFD group. (PDF) [file pone.0186216.s010.pdf]

S10 Table. Relative abundance of the 82 OTUs responding to oil treatment identified by redundancy analysis (RDA). Data are presented as the means ± S.D and analyzed by Mann-Whitney test, ****P*<0.001, ***P*<0.01 and **P*<0.05 *vs* the HFD group.

OUT	Control （%）	HFD (%)	HFD+M (%)	HFD+FO600 (%)	HFD+KO600 (%)	HFD+FO300KO300 (%)	HFD+FO400KO200 (%)	HFD+FO450KO150 (%)
OTU2708	1.381±0.047***	0.004±0.001	0.653±0.024**	0.003±0.001	0.006±0.002	0.000±0.000	0.000±0.000	0.042±0.011*
OTU14	1.044±0.013***	0.004±0.001	0.122±0.036*	0.019±0.001	0.000±0.000	1.517±0.431***	0.015±0.006	0.011±0.004
OTU135	2.705±0.064***	0.011±0.004	0.158±0.047*	0.003±0.001**	0.069±0.012*	1.094±0.179***	0.030±0.012*	0.025±0.009*
OTU149	0.774±0.110***	0.007±0.002	0.003±0.001	0.000±0.000	0.715±0.132***	0.101±0.065	0.009±0.001	0.616±0.077***
OTU36	0.273±0.018***	0.004±0.002	0.044±0.012**	0.000±0.000	0.000±0.000	0.000±0.000	0.000±0.000	0.002±0.001
OTU42	2.409±0.206***	0.036±0.012	0.199±0.031**	0.048±0.012	0.013±0.032	0.124±0.029**	0.310±0.101**	0.789±0.145***
OTU45	1.077±0.209***	0.021±0.011	4.225±1.121***	0.044±0.011*	0.142±0.034*	3.453±1.318***	0.513±0.032**	0.164±0.046*
OTU136	0.607±0.018 ***	0.018±0.002	0.096±0.033*	0.022±0.003	0.016±0.004	0.203±0.067**	0.169±0.056**	0.246±0.077**
OTU191	0.368±0.121***	0.011±0.002	0.000±0.000	0.000±0.000	0.000±0.000	0.000±0.000	0.055±0.007*	0.002±0.001
OTU81	0.114±0.006***	0.004±0.001	0.000±0.000	0.000±0.000	0.003±0.001	0.055±0.019*	0.000±0.000	0.002±0.001
OTU56	0.091±0.017**	0.004±0.002	0.000±0.000	0.016±0.021	0.019±0.003	0.014±0.007	0.000±0.000	0.000±0.000
OTU128	0.095±0.010**	0.004±0.001	0.085±0.023**	0.003±0.001	0.016±0.002*	0.029±0.011*	0.000±0.000	0.067±0.011**
OTU6298	0.082±0.023**	0.004±0.001	0.000±0.000	0.000±0.000	0.003±0.001	0.002±0.001	0.006±0.001	0.002±0.001
OTU475	0.084±0.024**	0.004±0.002	0.616±0.217***	0.006±0.002*	0.000±0.000*	0.040±0.004*	0.000±0.000*	0.110±0.023**
OTU92	1.082±0.327***	0.046±0.014	0.609±0.206**	0.388±0.121**	0.125±0.015**	1.366±0.198***	0.110±0.059**	0.226±0.058**
OTU97	0.083±0.013**	0.004±0.001	0.107±0.048***	0.064±0.012**	0.457±0.143***	0.710±0.119***	0.479±0.121***	0.540±0.146***
OTU179	0.154±0.015***	0.007±0.002	0.328±0.076***	0.082±0.023**	0.162±0.045***	0.023±0.012**	0.058±0.011*	0.025±0.004*
OTU304	0.075±0.009**	0.004±0.001	0.018±0.001*	0.022±0.003*	0.066±0.011*	0.002±0.001	0.009±0.002	0.019±0.006*
OTU8711	0.146±0.053**	0.007±0.003	0.000±0.000	0.000±0.000	0.033±0.021*	0.139±0.065***	0.006±0.001	0.005±0.001
OTU562	0.067±0.033**	0.004±0.001	0.051±0.012*	0.006±0.001	0.000±0.000	0.098±0.043*	0.076±0.021*	0.019±0.002
OTU335	0.903±0.207***	0.060±0.021	0.000±0.000*	0.025±0.012	0.036±0.021	0.008±0.001*	0.298±0.039**	0.107±0.031**
OTU409	0.058±0.018**	0.004±0.001	0.133±0.046***	0.000±0.000	0.069±0.024**	0.063±0.014**	0.012±0.004*	0.031±0.010**
OTU521	0.054±0.023**	0.004±0.001	0.003±0.001	0.022±0.002*	0.003±0.001	0.017±0.001	0.003±0.001	0.016±0.004
OTU342	0.109±0.076***	0.007±0.002	0.059±0.014*	0.000±0.000	0.119±0.021***	0.133±0.054***	0.147±0.026***	0.169±0.035***
OTU51	0.766±0.298**	0.053±0.012	0.369±0.101**	0.0191±0.045*	0.132±0.021*	0.200±0.100*	0.113±0.038*	0.212±0.043*
OTU174	0.156±0.026**	0.010±0.003	0.022±0.003	0.016±0.023	0.049±0.021	0.092±0.012*	0.003±0.001	0.512±0.154**
OTU142	0.453±0.021**	0.031±0.010	0.280±0.076**	0.012±0.004	0.062±0.003	0.191±0.012**	0.036±0.012	0.019±0.005
OTU1084	0.103±0.018**	0.007±0.001	0.000±0.000	0.000±0.000	0.069±0.023*	0.174±0.025**	0.006±0.001	0.028±0.011*
OTU440	0.054±0.023*	0.004±0.001	0.063±0.015*	0.019±0.021	0.000±0.000	0.052±0.012*	0.033±0.001*	0.016±0.004
OTU13	0.412±0.078**	0.031±0.009	0.048±0.013	0.480±0.102	0.125±0.054**	0.060±0.004*	0.089±0.021*	0.082±0.013*
OTU161	0.218±0.097**	0.017±0.007	0.052±0.011*	0.073±0.021*	0.006±0.001*	0.174±0.034**	0.027±0.004	0.022±0.001
OTU762	0.085±0.025*	0.007±0.001	0.000±0.000	0.000±0.000	0.026±0.006*	0.003±0.001	0.015±0.002	0.002±0.001
OTU178	0.293±0.056**	0.028±0.012	0.487±0.112***	0.470±0.121***	0.046±0.013*	0.133±0.056**	0.043±0.002*	0.036±0.009*
OTU146	0.048±0.012*	0.004±0.001	0.004±0.001	0.003±0.001	0.000±0.000	0.26±0.012*	0.000±0.000	0.002±0.001
OTU147	0.044±0.009**	0.004±0.001	0.048±0.012**	0.209±0.101***	0.013±0.003*	0.023±0.014*	0.101±0.034***	0.090±0.023**
OTU388	0.048±0.002*	0.004±0.002	0.004±0.001	0.060±0.021*	0.029±0.011	0.014±0.003	0.003±0.001	0.107±0.048**
OTU642	0.044±0.022**	0.004±0.001	0.026±0.007*	0.006±0.001	0.000±0.000	0.052±0.017**	0.000±0.000	0.065±0.017**

OTU16	1.463±0.768***	0.142±0.046	0.631±0.127**	0.845±0.286**	0.830±0.108**	0.710±0.190**	0.055±0..010*	1.519±0.466***
OTU470	0.072±0.023*	0.007±0.003	0.011±0.004	0.000±0.000	0.013±0.003	0.000±0.000	0.046±0.010*	0.000±0.000
OTU16301	0.073±0.018*	0.007±0.002	0.000±0.000	0.000±0.000	0.000±0.000	0.000±0.000	0.000±0.000	0.000±0.000
OTU158	0.172±0.023**	0.018±0.005	0.007±0.001*	0.019±0.012	0.000±0.000*	0.011±0.001	0.018±0.006	0.025±0.008
OTU478	0.104±0.029***	0.011±0.002	0.185±0.056***	0.000±0.000*	0.013±0.001	0.182±0.002***	0.061±0.009**	0.008±0.001
OTU724	0.103±0.018***	0.011±0.002	0.026±0.002	0.003±0.001	0.000±0.000	0.040±0.010**	0.049±0.021**	0.002±0.001
OTU321	0.105±0.021**	0.011±0.001	0.078±0.014**	0.028±0.014	0.023±0.012	0.063±0.010**	0.043±0.011*	0.045±0.014*
OTU522	0.037±0.006*	0.004±0.001	0.089±0.021**	0.000±0.000	0.003±0.001	0.000±0.000	0.000±0.000	0.000±0.000
OTU769	0.033±0.001**	0.004±0.001	0.074±0.021***	0.000±0.000	0.003±0.001	0.017±0.002*	0.003±0.001	0.016±0.003*
OTU112	0.006±0.001*	0.060±0.010	0.022±0.006*	0.003±0.001*	0.218±0.057***	0.011±0.001*	0.000±0.000*	0.147±0.026***
OTU636	0.007±0.001**	0.060±0.014	0.000±0.000**	0.006±0.001**	0.006±0.001**	0.008±0.001**	0.021±0.006*	0.011±0.005*
OTU839	0.003±0.002**	0.032±0.010	0.002±0.001**	0.003±0.001**	0.013±0.004	0.017±0.011*	0.021±0.011*	0.006±0.002**
OTU3534	0.003±0.001**	0.032±0.005	0.000±0.000**	0.000±0.000*	0.006±0.001**	0.000±0.000**	0.015±0.002*	0.008±0.002**
OTU397	0.013±0.004***	0.135±0.062	0.004±0.001***	0.006±0.002***	0.089±0.021**	0.017±0.001***	0.003±0.001***	0.093±0.016**
OTU366	0.007±0.002**	0.067±0.013	0.004±0.001**	0.016±0.002**	0.013±0.002**	0.011±0.001**	0.030±0.005*	0.019±0.011**
OTU3624	0.013±0.007**	0.139±0.052	0.011±0.004**	0.003±0.001***	0.016±0.013**	0.029±0.012**	0.039±0.006**	0.000±0.000***
OTU190	0.023±0.011***	0.249±0.027	0.007±0.002***	0.064±0.012***	0.079±0.021***	0.034±0.002***	0.153±0.014**	0.067±0.017***
OTU153	0.009±0.002***	0.107±0.044	0.476±0.127***	0.461±0.112***	0.205±0.034*	0.269±0.102*	0.439±0.245***	0.113±0.043
OTU3681	0.003±0.001	0.004±0.001	0.000±0.000	0.000±0.000	0.006±0.002	0.005±0.001	0.015±0.002	0.008±0.001
OTU288	0.010±0.007*	0.117±0.002	0.004±0.001**	0.133±0.043	0.046±0.012*	0.087±0.014	0.485±0.113**	0.206±0.058*
OTU433	0.007±0.002*	0.078±0.025	0.000±0.000*	0.003±0.001*	0.089±0.013	0.031±0.010*	0.006±0.001*	0.031±0.014*
OTU487	0.003±0.001***	0.039±0.012	0.004±0.001***	0.009±0.001***	0.009±0.002***	0.020±0.001*	0.030±0.004	0.014±0.004**
OTU763	0.003±0.001**	0.039±0.011	0.000±0.000**	0.000±0.000**	0.003±0.001**	0.034±0.012	0.000±0.000	0.033±0.011
OTU87	0.050±0.001**	0.664±0.112	0.055±0.010**	1.064±0.327**	0.917±0.231*	0.026±0.010***	0.654±0.067	0.534±0.276
OTU555	0.006±0.005**	0.089±0.013	0.000±0.000**	0.009±0.002**	0.029±0.005**	0.020±0.010**	0.049±0.012*	0.025±0.014**
OTU11205	0.003±0.003*	0.046±0.010	0.000±0.000*	0.016±0.003*	0.000±0.000*	0.000±0.000*	0.000±0.000*	0.000±0.000*
OTU314	0.007±0.004*	0.010±0.001	0.007±0.001*	0.015±0.002*	0.036±0.003**	0.043±0.019**	0.070±0.003***	0.028±0.001**
OTU346	0.007±0.002***	0.103±0.010	0.059±0.013**	0.003±0.001***	0.066±0.021**	0.011±0.001***	0.012±0.006***	0.005±0.001***
OTU15	0.060±0.005***	1.045±0.522	0.137±0.055***	2.777±0.476***	6.680±1.265***	0.165±0.047***	1.686±0.244*	0.531±0.012**
OTU109	0.007±0.007**	0.150±0.037	0.000±0.000**	0.006±0.001**	0.000±0.000**	0.000±0.000**	0.000±0.000**	0.000±0.000**
OTU6	0.206±0.109***	5.291±1.021	3.248±1.110***	1.001±0.003***	4.552±1.210*	2.147±1.001***	2.658±0.345***	4.056±1.241*
OTU345	0.003±0.001**	0.092±0.010	0.003±0.001**	0.025±0.010**	0.019±0.004**	0.023±0.010**	0.046±0.021**	0.019±0.004**
OTU381	0.007±0.003**	0.213±0.018	0.003±0.001***	0.231±0.102	0.129±0.065*	0.049±0.012**	0.460±0.110**	0.113±0.025*
OTU106	0.013±0.006***	0.451±0.101	0.321±0.109*	1.001±0.243***	0.334±0.028*	0.484±0.121	2.009±0.134***	1.210±0.234***
OTU25	0.047±0.012***	1.695±0.201	0.303±0.078***	1.176±0.453*	1.615±0,657	0.745±0.134***	1.299±0.456*	1.502±0.329*
OTU284	0.007±0.001***	0.263±0.019	0.004±0.001***	0.003±0.001***	0.013±0.006***	0.029±0.010***	0.046±0.017***	0.014±0.002***
OTU124	0.003±0.001**	0.163±0.017	0.554±0.107***	0.000±0.000***	0.152±0.035***	0.621±0.140***	0.909±0.143***	0.311±0.067***
OTU207	0.003±0.001**	0.174±0.010	0.011±0.002**	0.162±0.045	0.125±0.021	0.058±0.021**	0.184±0.078	0.684±0.043**
OTU3	0.142±0.057***	9.147±1.287	0.133±0.026***	0.479±0.104***	7.753±1.237*	2.545±0.789***	4.176±0.759**	3.089±0.987**
OTU285	0.003±0.001***	0.238±0.027	0.018±0.003***	0.009±0.001***	0.013±0.002***	0.017±0.010***	0.000±0.000***	0.025±0.002***
OTU33	0.010±0.001***	0.824±0.198	0.295±0.023***	0.896±0.120	0.486±0.103***	0.003±0.001***	0.503±0.101***	0.257±0.049***
OTU39	0.023±0.011***	2.057±0.765	0.314±0.100***	1.630±0.453*	1.754±0.865**	0.481±0.101***	0.811±0.165***	1.072±0.045***

OTU29	0.063±0.026***	9.069±1.906	0.066±0.022***	4.894±1.124***	2.592±0.769***	0.148±0.024***	13.222±2.876**	0.905±0.032***
OTU91	0.003±0.001***	1.442±0.576	0.343±0.132***	0.972±0.120*	1.125±0.119	0.496±0.101***	1.210±0.117	2.690±0.489**
OTU22	0.007±0.004***	3.214±0.769	2.366±0.101*	7.960±1.345***	0.629±0.123***	3.424±1.011	6.124±0.989**	0.079±0.018***
