# Supplementary figures and images for: Modulation of the gut microbiota by the mixture of fish oil and krill oil in high-fat diet-induced obesity mice
Source: PLoS One. 2017 Oct 9;12(10):e0186216. doi: 10.1371/journal.pone.0186216 (PMC5633193; doi:10.1371/journal.pone.0186216)

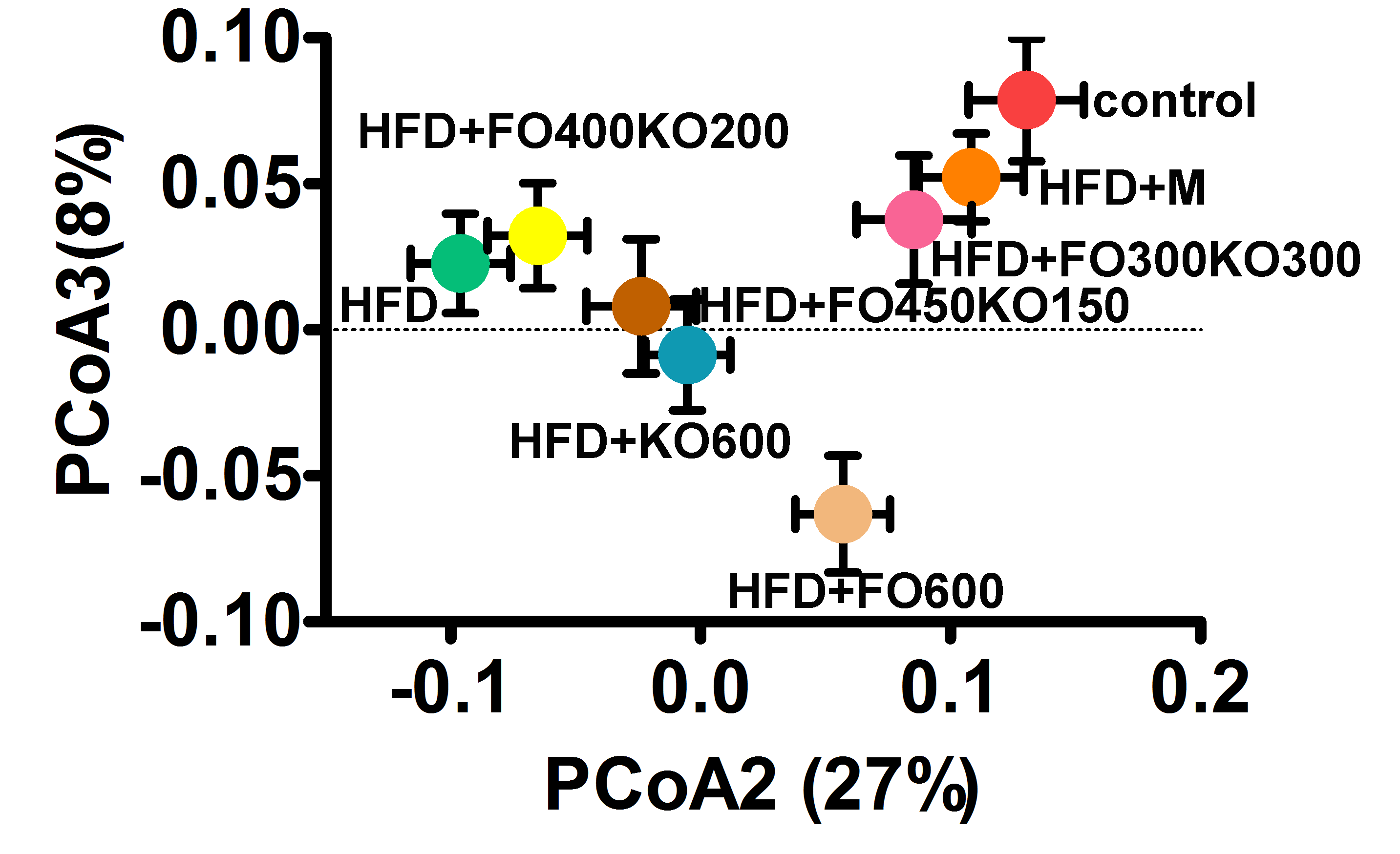

Supplement: S1 Fig — Data are presented as the means ± S.D. Each point represents the mean principal coordinate (PC) score of all mice in a group, and the error bar represents the S.D. (TIF) [file pone.0186216.s011.tif]

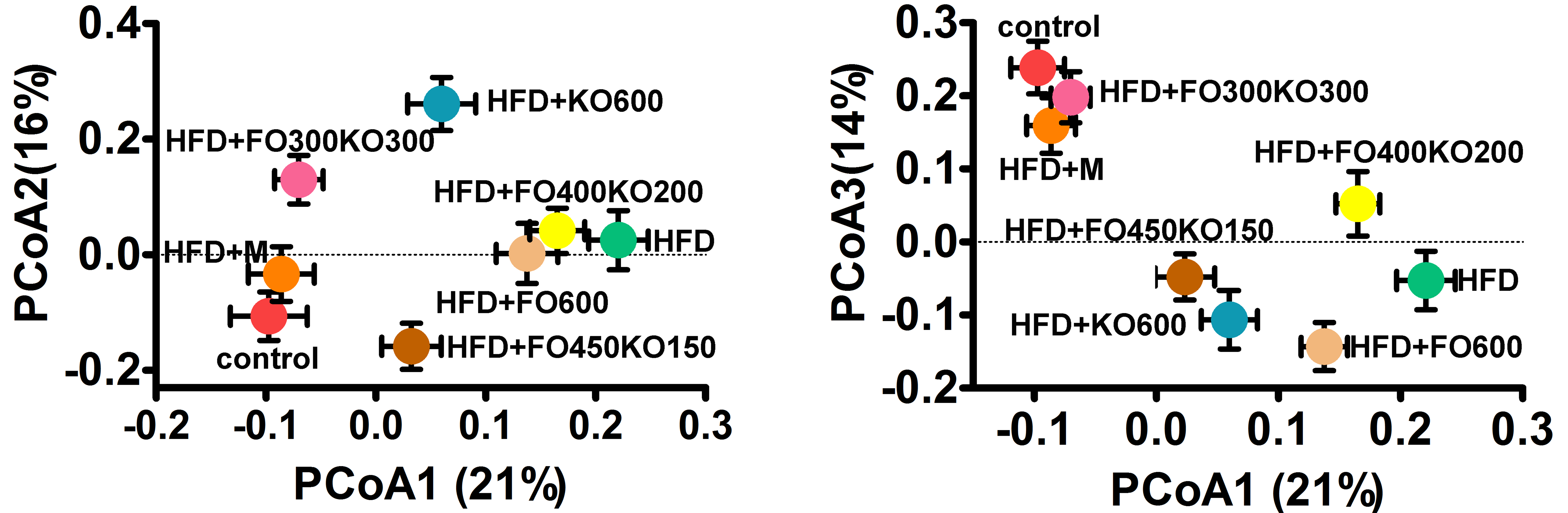

Supplement: S2 Fig — Data are presented as the means ± S.D. Each point represents the mean principal coordinate (PC) score of all mice in a group, and the error bar represents the S.D. (TIF) [file pone.0186216.s012.tif]
